# Illuminating the newly produced viruses within the virosphere with bioorthogonal noncanonical amino acid tagging and single-virus genomic sequencing technologies

**DOI:** 10.1093/ismeco/ycag048

**Published:** 2026-03-06

**Authors:** Maria Alvarez-Sanchez, Francisco Martinez-Hernandez, Aitana Llorenç Vicedo, Marina Vila-Nistal, Alon Philosof, Aditi K Narayanan, Jamie C Tijerina, Oscar Fornas, Manuel Martinez-Garcia, Victoria J Orphan

**Affiliations:** Department of Physiology, Genetics, and Microbiology, University of Alicante, Carretera San Vicente del Raspeig, Alicante 03690, Spain; Multidisciplinary Institute for Environmental Studies (IMEM), University of Alicante, Carretera San Vicente del Raspeig, Alicante 03690, Spain; Department of Physiology, Genetics, and Microbiology, University of Alicante, Carretera San Vicente del Raspeig, Alicante 03690, Spain; Division of Biology and Biological Engineering, California Institute of Technology, Pasadena, CA 91125, United States; Department of Physiology, Genetics, and Microbiology, University of Alicante, Carretera San Vicente del Raspeig, Alicante 03690, Spain; Multidisciplinary Institute for Environmental Studies (IMEM), University of Alicante, Carretera San Vicente del Raspeig, Alicante 03690, Spain; Department of Physiology, Genetics, and Microbiology, University of Alicante, Carretera San Vicente del Raspeig, Alicante 03690, Spain; Division of Biology and Biological Engineering, California Institute of Technology, Pasadena, CA 91125, United States; Division of Biology and Biological Engineering, California Institute of Technology, Pasadena, CA 91125, United States; Caltech Flow Cytometry Facility, California Institute of Technology, Pasadena, CA 91125, United States; Centre for Genomic Regulation (CRG), The Barcelona Institute for Science and Technology (BIST), Carrer del Doctor Aiguader, 88, PRBB Building, Barcelona 08003, Spain; Flow Cytometry Unit, Pompeu Fabra University (UPF) and Centre for Genomic Regulation (CRG), Carrer del Doctor Aiguader, 88, 08003 Barcelona, Spain; Department of Physiology, Genetics, and Microbiology, University of Alicante, Carretera San Vicente del Raspeig, Alicante 03690, Spain; Multidisciplinary Institute for Environmental Studies (IMEM), University of Alicante, Carretera San Vicente del Raspeig, Alicante 03690, Spain; Division of Biology and Biological Engineering, California Institute of Technology, Pasadena, CA 91125, United States; Division of Geological and Planetary Sciences, California Institute of Technology, Pasadena, CA 91125, United States

**Keywords:** BONCAT, virus, bacteria, FACS, single-virus genomics, Far-T4 phages, OM43 phages, pelagiphage, OM43, NCLDV

## Abstract

Marine viruses impact biogeochemical cycles through cell lysis, releasing organic matter and nutrients that fuel ocean productivity. Identifying and quantifying the specific viruses active in these processes remain a priority in the field. Here, we introduce a click-chemistry method to fluorescently label, sort, and sequence the genomes of newly produced viral particles (viral progeny) released from transcriptionally active host microbial cells, alongside the analysis of co-occurring inactive cells and pre-existing viruses in environmental samples. This approach, called viral bioorthogonal noncanonical amino acid tagging (BONCAT)-fluorescence-activated cell sorting (FACS), combines BONCAT with environmental sample incubation, followed by single-virus and single-cell sorting by flow cytometry (FACS). Genomic analysis of translationally active cells and new viral progeny in coastal seawater incubations confirmed BONCAT labeling and successful sorting of diverse marine bacteria, microeukaryotic cells, and virioplankton, with stark differences in the predicted turnover of specific groups of infecting viruses, including pelagiphages, methylophages, a Flavobacteriales-associated novel “Far-T4” clade, noncanonical DNA viruses of Naomiviridae using dU instead of dT, algae-infecting giant NCLDV viruses, and parasitic virophages. Sequenced BONCAT-active cells showed a strong enrichment in viral contigs relative to the inactive cell fraction, suggestive of a large proportion of translationally active virocells. This study illustrates the effectiveness of viral BONCAT-FACS for uncovering genome-resolved virus–host dynamics. By providing a direct approach for tracking active viral infections in natural environments, this method enhances our ability to investigate behavior and interactions of these nanoscale predators, expanding our understanding of their role in ecosystem dynamics.

## Introduction

**Figure 1:**
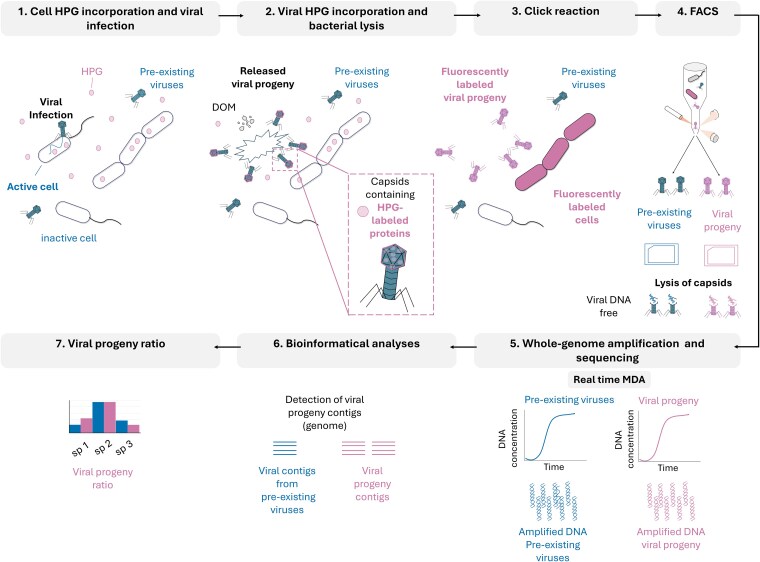
Schematic workflow of the viral BONCAT-FACS methodology for detecting viral progeny and pre-existing viruses in natural marine microbial assemblages. Diagram showing the steps involved in the viral BONCAT-FACS approach, coupled with downstream genomic analyses: (i) microbial cell HPG incorporation and viral infection. Environmental samples are incubated with HPG, which is incorporated into *de novo* synthesized proteins of active microbial cells. During this period, pre-existing and infecting virions remain indistinguishable. (ii) Viral HPG incorporation and microbial cell lysis. In actively infected cells, HPG is incorporated into newly synthesized capsid proteins and lysis releases BONCAT-positive virions (viral progeny), which contribute to host mortality and DOM release. (iii) Click-reaction: BONCAT-tagging enables fluorescence labeling of both active cells and novel viral progeny based on HPG incorporation. (iv) FACS: sorting of viral progeny and pre-existing viruses. BONCAT-positive and -negative viral populations are separated by FACS. (v) Capsid lysis and whole-genome amplification using real-time MDA. (vi) Bioinformatic analyses: sequencing and assembly facilitate the identification of genomes from viral progeny and pre-existing viral contigs. (vii) Viral progeny ratio: turnover of viral populations is assessed by the ratio of BONCAT-positive viruses to total viral abundance, which informs on viral production and contribution to DOM in the ecosystem.

Viruses, including bacteriophages (herein referred to as viruses), are the most abundant biological entities in nature, exerting a significant impact across all ecosystems by influencing biogeochemical cycles [1, 2], shaping genetic composition of host cells, and modulating microbial abundance through viral lysis [3–5]. After a successful viral infection, microbial cells (particulate organic matter) are transformed into cell debris, releasing dissolved organic matter (DOM), that fuels ocean productivity. This process, known as the viral shunt [6], recycles up to 50% of photosynthetically fixed carbon, an estimated 10 billion tons annually, in global ocean ecosystems [1, 4, 7]. During cell lysis, the viral progeny (the newly produced viral particles) are released into the environment, renewing the virosphere, which is likely variable in both the rate of infection and burst size for different virus–host pairings (Fig. 1). These factors, alongside the decay of viral particles, contribute to viral turnover [8]. Methodological limitations currently restrict our ability to quantify viral turnover and residence times at local scales, resulting in poorly resolved ecological dynamics [2, 9].

Traditional methods, such as environmental sample incubation with radiolabeled leucine or thymidine substrates [10], have been used to assess viral production, but these techniques lack information about the identity of the newly produced viral progeny. Conversely, viromics and microbial metagenomics provide information about the total diversity, biogeography, relative abundance of viruses, and predicted virus–host interactions but do not discriminate between the newly produced virions and the resilient pre-existing free populations within the viral assemblage [11, 12]. Several studies have additionally employed metatranscriptomics to assess expression of genes indicative of viral infections within microbial cells as an estimate for viral lysis [13–17]. In addition, other host- and virus-targeted RNA approaches, such as single-cell transcriptomics [18, 19] or messenger RNA-targeted fluorescence in situ hybridization (mRNA-FISH) [20], have been used to detect strong viral transcriptional activity and infections at the cellular level. These approaches can be challenging, however, as the proportion of viral reads in cellular metatranscriptomic libraries is low, and transcripts often cannot differentiate between successful lytic viral production and abortive infections that do not result in cell mortality and new viral particles. Other methodologies employing DNA-based stable isotope probing from concentrated virus-like particles (VPSs) in sample incubations have also shown promise for tracking newly produced virions [21–23]; however, this technique requires large sample volumes and does not enable tracking at the level of individual viral particles. The current limitations in methodologies for directly identifying and quantifying newly produced viruses among total viral assemblages in the environment represent a bottleneck to advancing our understanding of critical ecological processes such as cell mortality, virus–host dynamics, and virus-mediated nutrient cycling.

To address this shortcoming, we developed a synergistic methodological approach that combines protein targeted bioorthogonal noncanonical amino acid tagging (BONCAT) for fluorescent labeling of newly produced viral progeny (viral BONCAT) [24, 25] with viral particle sorting by flow cytometry (fluorescence-activated cell sorting, FACS) [26, 27] and single-virus genome sequencing technologies [11, 28, 29]. Employing the viral BONCAT-FACS method in tandem with earlier developed cellular BONCAT-FACS, we demonstrate the detection, quantification, and genomic characterization of diverse newly produced viral progeny from successful active viral infections in environmental incubations, differentiated from co-occurring non-active (BONCAT-negative) microbial cells and pre-existing virions in the sample. The BONCAT assay requires sample incubation with an azide or alkyne-modified methionine analogs l-azidohomoalanine (AHA) or l-homopropargyl glycine (HPG) added at low concentrations. These bioorthogonal amino acids have been shown to be readily incorporated by diverse microorganisms (archaea, bacteria, and microeukaryotes) [30] during protein synthesis using the cell’s native translational machinery, and AHA or HPG-labeled proteins are subsequently conjugated to a fluorophore using Cu(I)-catalyzed azide-alkyne cycloaddition (i.e. click chemistry [24]). Applications of BONCAT (and BONCAT-FACS) in environmental microbial ecology studies (e.g. [30]) have provided insights into the physiology and genomic diversity of active uncultured members of microbial communities from diverse ecosystems, ranging from deep ocean sediments to hot springs, using incubation conditions mimicking the native environment, or used to track the activity of select community members in response to a stimulus [27, 30–38]. Very recently, BONCAT data demonstrated that this approach is effective to estimate metabolic activity correlating perfectly with standard radioisotope-based techniques across different ocean depth gradients [39]. BONCAT has also been shown to label newly produced free virions after infection of a translationally active microbial host using epifluorescence microscopy [25] as well as used to detect labeled viral proteins with proteomics methods in defined co-cultures [25, 40]. These studies provided evidence supporting the applicability of this protein-based labeling approach to viral systems, with clear potential for virus ecology research. Here we advance our earlier developed microscopy-based viral BONCAT method [25] for use with higher-throughput flow cytometry combined with single-virus sequencing technologies. With viral BONCAT-FACS, we demonstrate the ability to simultaneously sort and genomically characterize discrete populations of newly produced viruses and co-occurring active and inactive microbial cells spanning large size range and fluorescence intensities in the sample. This methodological approach assists with deconvolving complex virus–host interactions in environmental samples, discriminating between newly produced (lytic) viral clades with likely ecological impact, as opposed to pre-existing viral populations with slow decay rates. Importantly, as viral BONCAT-FACS is based on labeled proteins (e.g. capsids) rather than nucleic acids, this methodological approach should in principle be able to capture both DNA and RNA viruses and can identify them independent of whether related genome sequences are previously known. We demonstrate the efficacy of the viral BONCAT-FACS method coupled with single-virus and cell sequencing technologies for investigating complex communities in environmental samples, here characterizing paired newly produced viruses in contrast to those pre-existing dsDNA viruses in the sample as well as microbial communities in coastal seawater incubations from the Pacific Ocean and Mediterranean Sea.

## Materials and methods

### Sample collection and processing

Freshly collected coastal surface seawater samples from the Mediterranean Sea (Cape the Huertas, Alicante, Spain; 38° 21′ 14.3″ N, 0° 25′ 36.6″ W; 9 November 2023) and the Pacific Ocean (Corona del Mar, California, USA, 33° 35′ 47.6″ N, 117° 52′ 48.9″ W; 29 April 2023) were used for BONCAT experiments. The average water temperature for these sites was 18°C during the respective months. A total of 2 l (Mediterranean) and 0.25 l (Pacific) of seawater were each amended with 10 μM l-homopropargylglycine (HPG; ref. CLK-1067-100, Jena Bioscience) and incubated for 5 days at 18°C under light:dark photoperiods of 12 h:12 h (Mediterranean) and 14 h:10 h (Pacific), allowing for the assimilation of HPG into proteins within translationally active microbial cells and their infecting viruses (Fig. 1, Supplementary Fig. 1). Parallel control incubations of the same volume without HPG were used to establish fluorescence thresholds in flow cytometry (FACS) analyses. These thresholds allowed us to discriminate active cells and newly produced virus populations from inactive microbial and pre-existing viral populations in HPG-incubated samples during subsequent fluorescence-based analyses. Samples from the Mediterranean were subjected to two different click reaction conditions: (i) using the standard 30 min click and (ii) click incubation for 6 h, while samples from the Pacific each used a 6 h click reaction. The shorter reaction followed the published BONCAT protocol for cells, while the extended 6 h reaction was developed here to enhance the fluorescence signal and differentiation of BONCAT-positive viral populations. After incubation, seawater was concentrated using a tangential flow filtration system (Vivaflow 30 000 MWCO PES cassettes; Vivaflow 200, Sartorius), followed by further concentration until a final volume of ~15–20 ml using Amicon Ultra Centrifugal filters 100 kDa (Amicon Ultra, Merck Millipore). Sample concentrates were washed 3× with 0.02 μm-filtered PBS (pH = 7.4) to a final volume of ~208 μl and kept at 4°C to minimize cell and viral decay until sorting (within 12 h). Further details are provided in Supplementary Methods and in Supplementary Fig. 1.

### Bioorthogonal noncanonical amino acid tagging-click chemistry reaction stain of active populations

Active microbial cells and viral progeny were fluorescently labeled using a solution-based BONCAT protocol [25, 27, 30] optimized for viral BONCAT detection in FACS while preserving nucleic acids for downstream genome recovery (full experimental details are provided in the Supplementary Methods and in Supplementary Fig. 1). Briefly, 208 μl of concentrated samples were incubated in PCR tubes with 42 μl of freshly prepared click reaction cocktail containing CuSO₄ (0.5 mM, Sigma-Aldrich), THPTA (2.5 mM, Jena Bioscience), AF647-Picolyl-azide (0.1 μM, Jena Bioscience), aminoguanidine (25 mM, Sigma-Aldrich), and sodium ascorbate (25 mM, Sigma-Aldrich) in PBS buffer (final concentrations). To minimize oxygen interference, all reagents and buffers were prebubbled with argon prior to preparation. Click reactions were performed at room temperature in the dark for either 30 min or 6 h. Following labeling, samples were washed and concentrated with 100-kDa Amicon Ultra Centrifugal filters (Merck Millipore) to remove excess dye. Pacific Ocean samples were additionally counterstained with SYBR™ Gold (Thermo Fisher Scientific) to visualize total DNA-containing cells and viruses. All samples were kept at 4°C and analyzed by FACS within the same day or in <12 h.

### Fluorescence microscopy protocol for click reaction with different incubation times

**Figure 2:**
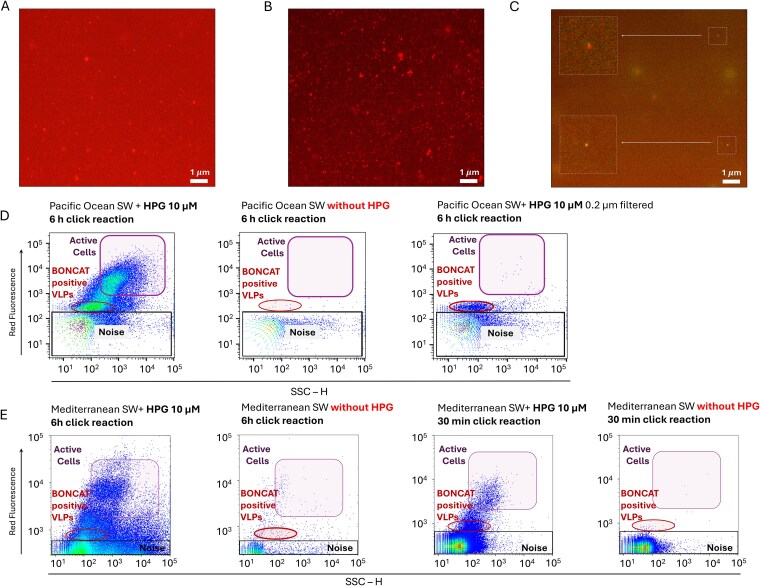
Detection and identification by viral BONCAT-FACS of newly synthesized viruses in coastal seawater from the Mediterranean Sea and the coastal Pacific Ocean. (A) Fluorescence microscopy image showing viral progeny from a Pacific seawater sample after a 30-min click reaction using the standard viral BONCAT protocol with AF647-pycolyl-azide (red fluorescence). The high background signal prevents reliable BONCAT+ virus discrimination by FACS in this condition. (B) Fluorescence microscopy image after an extended (6-h) click reaction with the optimized viral BONCAT protocol for cytometry, showing reduced background fluorescence and improved signal-to-noise ratio. Images were collected with the same exposure setting. (C) Overlap epifluorescence image of sorted BONCAT-labeled VLPs from the viral progeny fraction. Magnified regions highlight representative viral particles double-stained with AF647-pycolyl-azide and SYBR-gold. Scale bars for panels A–C represent 1 μm. (D) Flow cytometry plots of Pacific Ocean samples following a 6-h click reaction. BONCAT-positive active cells and viral progeny are distinguished by fluorescence, compared to a seawater control incubated without HPG, which defines the threshold for sorting. In order to detect the seawater sample with HPG by FACS, it was filtered through 0.2 μm to better resolve the viral BONCAT-positive population. (E) Flow cytometry plots for Mediterranean samples after 6-h and 30-min click reactions. Both HPG-incubated samples show BONCAT-positive cells and viral progeny, compared to corresponding controls without HPG at each click-reaction time, used to set thresholds for BONCAT-negative events. For both (D) and (E): *x*-axis represents SSC-H (side scatter, relative units); *y*-axis represents fluorescence from AF647-pycolyl-azide in relative units.

Fluorescence microscopy was performed on 500 ml seawater samples from the Pacific Ocean incubated with 10 μM HPG in an independent replicate experiment. Samples were concentrated and washed with 0.02-μm-filtered 1× PBS using 100-kDa Amicon centrifugal filters (7500 × g, 3 min). The concentrate was divided into two equal aliquots for click reaction incubations of 30 min and 6 h. In parallel, 500 ml seawater without HPG was processed as a negative control. Following the click reaction, samples were washed once with 500 μl filtered PBS, resuspended in 250 μl PBS, and counterstained with 1.25 μl of 1000× SYBR Gold for 20 min in the dark. Samples underwent three additional wash steps with 500 μl filtered PBS using centrifugal filtration and were finally resuspended in 500 μl PBS. Half of each sample (250 μl) was filtered through 0.02-μm Anodisc filters (Whatman), air-dried, and mounted with antifade medium. Samples were visualized with an Olympus BX51TRF epifluorescence microscope equipped with a Plan Apo 100× (NA 1.4) objective and an X-Cite 120Q excitation light source. Filter sets used were Chroma FITC/Alexa Fluor 488 (excitation: 480/40 nm, emission: 535/50 nm) for SYBR Gold, and Cy5 (excitation: 647/30 nm, emission: 670/30 nm) for AF647. Increasing the click reaction time from 30 min to 6 h improved the fluorescence signal-to-noise ratio (Fig. 2A and B).

### Fluorescence-activated cell sorting

FACS was performed using BD FACSAria™ Fusion (Becton Dickinson, San Jose, CA) instruments at Caltech (USA) and SCSIE, University of Valencia (Spain). Instruments, sheath fluid, and consumables were rigorously decontaminated to minimize DNA carryover, following protocols from single-cell genomics workflows [11, 29, 41–43] (see Supplementary Methods). Active microbial cells and newly produced viral particles were detected via BONCAT labeling (AF647-Picolyl-azide; Abs/Em = 648/671 nm) using the 640 nm red laser, while total populations were visualized with SYBR™ Gold (Abs/Em = 495/537 nm) using the 488 nm blue laser. Both fluorescent channels were represented versus the side scatter height (SSC-H) to facilitate population discrimination. Sorting was performed in bulk under single-cell mode for maximal purity, and sorted populations were collected into 96-well plates or ultraviolet sterilized tubes and then stored at −80°C until whole-genome amplification (WGA). Up to five pool replicates of BONCAT-positive populations from Mediterranean samples were sorted in 96-well plates (1000 and 5000 viruses from viral progeny; 200 active cells) using a flow rate of 40–800 ev/s. For the Pacific samples, each BONCAT-positive and BONCAT-negative population were sorted into a single pool in 16 h UV-irradiated 1.5 ml tubes [11, 41, 43] (100 000 viruses from viral progeny, 50 000 pre-existing viruses and 25 000 active and non-active microbial cells) and divided in wells before WGA from 96-well plate with 5000 viruses or cells each (20 wells for viral progeny, 10 wells for pre-existing viruses, and 5 wells for active and non-active cells). Furthermore, a total of 10 000 BONCAT-positive viruses from the Pacific Coast were sorted into a 1.5 ml Eppendorf tube and directly visualized by fluorescence microscopy using an Olympus BX51TRF epifluorescence microscope equipped with a Plan Apo 100× (NA 1.4) objective and an X-Cite 120Q excitation light source. The Chroma filter sets previously described were used to identify SYBR Gold and BONCAT (AF647+) overlapping signals (Fig. 2C). Detailed information on gating strategies, negative control samples, sorting criteria for each sample, and other details are provided in the Supplementary Methods.

### Whole-genome amplification

Sorted viral populations were lysed thermally (liquid nitrogen) and chemically with directed lysis buffer (DLB buffer; ref 1068797, Qiagen), while microbial cells underwent chemical lysis only. Genomic DNA from all lysed samples was then amplified using real-time monitored multiple displacement amplification (MDA) with EquiPhi29 polymerase (ref A39392, Thermo Fisher Scientific) [29], as described [44] (see Supplementary Material for more details). The MDA master mix contained 0.26 U of polymerase, 1× EquiPhi29 reaction buffer (Thermo Fisher Scientific), 0.04 mM heptamers (IDT), 10 mM DTT (Sigma), 0.4 mM deoxyribonucleotide triphosphate (dNTPs) (New England Biolabs), 0.002 μl SYTO 9 (Invitrogen), and UV-treated sterile MilliQ water. Positive controls consisted of lambda phage genomic DNA, while negative controls consisted of UV-treated 1× Tris-EDTA buffer (10 mM Tris, 1 mM EDTA; pH 8.0)) and sorted sheath fluid.

### Sequencing and assembly

MDA products were used to prepare Illumina libraries (Illumina DNA Prep (M) and adapters from IDT for Illumina DNA/RNA UD), following the manufacturer’s protocol. Illumina paired-end sequencing was performed with NovaSeq 6000 (150 × 2 PE; ~3 Gb/sample; Macrogen, Korea). Raw reads were quality-trimmed using Trimmomatic (v0.39) [45] and assembled using SPAdes (v3.15.5) [46]. Detailed parameters are provided in the Supplementary Methods.

### Taxonomic analysis of microbial reads

Trimmed reads were taxonomically classified with Kaiju [47] against the NCBI nr_euk database (including bacteria, archaea, eukaryotes, and viruses) and visualized with Krona [48]. To estimate the abundance of the bacterial clade OM43 in active and non-active microbial sorted fractions from the Pacific and the Mediterranean samples, trimmed reads were mapped against 13 publicly available OM43 isolate genomes from NCBI (e.g. HTCC2181 [49], KB13 [50], MBRSH7 [51], H5P1 [52], LSUCC series [53]) using BLASTn (BLAST 2.8.1+). Recruitment plots were generated using the Enveomics package [54]. A detailed description of parameters, thresholds, and filtering procedures is provided in the Supplementary Methods.

### Viral identification and bioinformatic analysis

Viral contigs were identified using a combination of VirSorter2.0 (v2.2.3) [55], CheckV (v0.9.0) [56], and geNomad (v1.7.0) [57]. Contigs were considered *bona fide* viruses if detected by multiple tools and confirmed via protein–protein BLAST (BLASTp 2.8.1+) against the IMG/VR v4 [58] reference database (sharing more than two proteins with ≥80% similarity and ≥ 90% coverage). See more details in Supplementary Methods. Specific viral groups found in the positive BONCAT fractions were further characterized using BLASTp comparisons as follows: OM43-like phages were identified by comparison with known isolates (Venkman [59], MEP301 [60], Melnitz [52], MEP401–402) and 99 related metagenomic genomes [61], while vSAG 37-F6 reference genome [11, 62] was used to detect similar viral contigs. Far-T4 phages were detected by screening viral BONCAT contigs >1500 bp for Gp23-like proteins [63]; all Gp23-like proteins (BLASTp bit score > 50, *e*-value <1 × 10^−10^) were aligned using MAFFT (v7.310) [64], trimmed using trimAI (v1.4) [65], and used to build phylogenetic trees using Geneious software [66]. Additionally, all proteins from viral contigs were BLASTp-compared against the proteins of Rhodothermus RM378 phage, the only isolated representative of Far-T4 phages, to detect core proteins. Giant viruses (Nucleocytoviricota) and virophages (Lavidaviridae) were identified among contigs >5000 bp detected with geNomad and manually verified using IMG/VR data to refine taxonomic annotation.

Protein-sharing networks were constructed using vConTACT2 (v0.9.19) [67] through the DOE Systems Biology Knowledgebase (Kbase, http://kbase.us) [68], with a Markov cluster algorithm (MCL) for protein clustering. The “Prokaryotic Viral RefSeq” was used as the reference database, and the network was visualized in Cytoscape (v3.7.1) [69]. Viral progeny ratios were calculated as the proportion of BONCAT-positive viruses relative to total viral populations within each cluster and normalized for initial sorting depth (further details for this parameter, see Supplementary Methods). Detailed workflows, parameters of each tool, and datasets are provided in the Supplementary Methods.

### Host assignment of the Far-T4-like phage cluster

Potential host prediction for the Far-T4-like phage cluster was based on protein-based comparisons combined with machine learning. Viral BONCAT proteins from the Far-T4-like cluster were screened against high-confidence genomes in the IMG/VR v4 [58] database using stringent BLASTp thresholds (≥80% shared proteins, ≥60% amino acid identity, ≥95% coverage), and the resulting IMG/VR matches were subsequently analyzed with iPHoP (v1.3.3) [70] to infer host associations (see Supplementary Methods for details).

## Results

### Optimizing viral bioorthogonal noncanonical amino acid tagging for flow cytometry sorting of environmental viruses

In the viral BONCAT assay, newly produced viruses are labeled during infection of an anabolically active (i.e. BONCAT-positive) microbial host, where the methionine analog AHA or HPG is directly incorporated into viral proteins derived from the host during the lytic cycle. Subsequently, free nanometer-scale viral progeny released after cell lysis can be directly visualized and identified by fluorescence labeling (Fig. 1, Supplementary Fig. 1).

The fluorescence intensity of BONCAT-positive environmental viruses using the original viral BONCAT protocol [25] was inadequate for FACS detection and sorting, as small particle detection by flow cytometry requires a stronger fluorescence signal compared with epifluorescence microscopy. Optimizing the click-chemistry protocol, we boosted the fluorescent labeling and demonstrated the ability to consistently detect VLPs above the background noise during flow cytometry sorting at two independent sorting facilities, illustrating its transferability between labs (Fig. 2, Supplementary Fig. 2) [11, 28, 29]. Specific protocol modifications for BONCAT included performing the click reaction under oxygen-free conditions using argon gas, as the copper (I) catalyzed click reaction is sensitive to oxygen [71] and dampens the fluorescence signal (detailed in Supplementary Methods; Supplementary Fig. 1). Additionally, the click reaction time was extended from the standard 30-min incubation to 6 h, which also substantially enhanced the fluorescence signal from individual VLPs (Fig. 2). Many of these variations were initially tested with a known culture model (*Escherichia coli* and phage T7) demonstrating the feasibility and validity of our viral BONCAT-FACS approach (Supplementary Fig. 3) and later implemented for environmental seawater samples.

**Figure 3:**
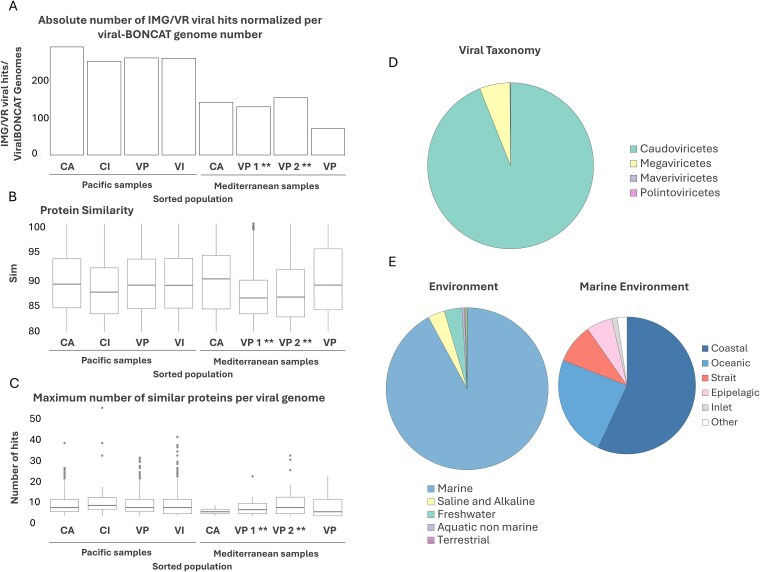
Sequencing results and genomic analysis of sorted viruses obtained by viral BONCAT-FACS. (A) Bar plot showing the mean number of protein BLAST hits per contig for viral BONCAT-FACS sorts from the Pacific Ocean (PO) and the Mediterranean Sea (MS) samples, grouped by sorted fraction: CA (cells active), CI (cells inactive), VP (viral progeny), and VI (pre-existing viruses). Mediterranean VP samples include two replicates of viral progeny after a 30-min click reaction (indicated by **). (B) Boxplot showing mean amino acid similarity of recovered viral BONCAT contigs to reference viral genomes. (C) Boxplot displaying the maximum number of similar proteins identified for viral BONCAT contigs against reference viral genomes. (D) Pie chart indicating the proportion of recovered viruses assigned to major taxonomic groups by geNomad. (E) Pie chart summarizing the source environments for the closest matching reference viral genomes to the recovered viral BONCAT-FACS sequences.

Incubation of Mediterranean and Pacific seawater with 10 μM HPG for 5 days under environmentally relevant conditions (detailed in Supplementary Methods) resulted in the incorporation of the analog into proteins of metabolically active microbes and their associated viruses (Fig. 1, Supplementary Fig. 1). From these two independent coastal seawater experiments, separate pools of positive BONCAT-labeled virions and cells, along with the co-occurring non-active cell and viral fractions (BONCAT negative), were sorted, whole-genome amplified [11, 29], and Illumina sequenced. From the Mediterranean Sea sample, 41,000 VLPs from the viral progeny assemblage (BONCAT-positive VLPs) and 3100 active microbial cells (BONCAT-positive cells) were sorted and sequenced. From the coastal Pacific Ocean BONCAT incubation, 100,000 VLPs from the viral progeny and 50,000 BONCAT negative VLPs were sorted and sequenced in addition to 25,000 BONCAT active and non-active microbial cells. Detection and corroboration of *bona fide* viral contigs from these sequencing datasets was performed using a combination of Virsorter 2.0 [55], geNomad [57], and compared against the IMG/VR [58] database v4.1 (using a protein similarity threshold of 80% and an alignment coverage of 90%; Fig. 3). Sequence datasets of the BONCAT-labeled viral fractions showed minimal contamination from cellular debris or outer membrane lipid vesicles, which have been shown to be common in seawater and are difficult to distinguish from viruses based on size and nucleic acid staining alone [72–75]. Epifluorescence microscopy was also used to independently confirm that the sorted viral progeny fraction indeed contained BONCAT-tagged and SYBR stained VLPs (Fig. 2C).

### Genomic characterization of newly produced virions within the virioplankton and lineage specific variation in viral production

**Figure 4:**
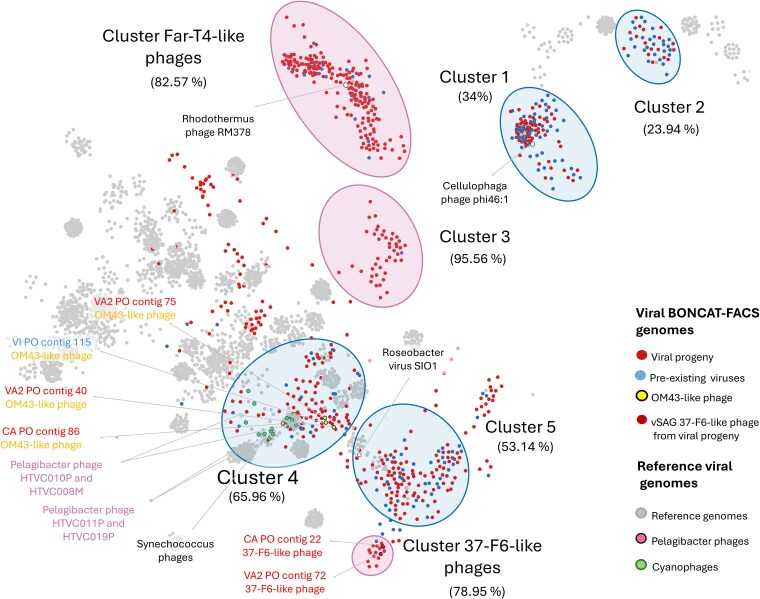
Protein cluster network of viral genome fragments obtained by viral BONCAT-FACS. Network visualization of viral genomes (>5 kb) recovered from the Mediterranean Sea (MS) and the Pacific Ocean (PO) viral BONCAT-FACS experiments. Nodes represent viral genomes, which are clustered based on shared protein similarity. Individual points indicate either newly produced viral progeny (BONCAT-positive) viruses or pre-existing viruses (BONCAT-negative) that persisted over the course of the incubation colored according to the legend. Points without color correspond to reference genomes from the Prokaryotic Viral RefSeq 201 database. Magenta ellipses highlight clusters dominated by viral progeny with little or no representation in the pre-existing (BONCAT-negative) viral fraction, suggesting high activity and rapid turnover of these viral populations. Blue ellipses mark clusters where the proportion of viral progeny (BONCAT-positive) was substantially less than the abundance of taxonomically related pre-existing viruses, reflecting populations with lower lytic activity and slower turnover rates. The calculated viral progeny ratio percentages for each cluster are provided in parentheses. Select reference genomes from isolated phages are highlighted and labeled in the network.

**Figure 5:**
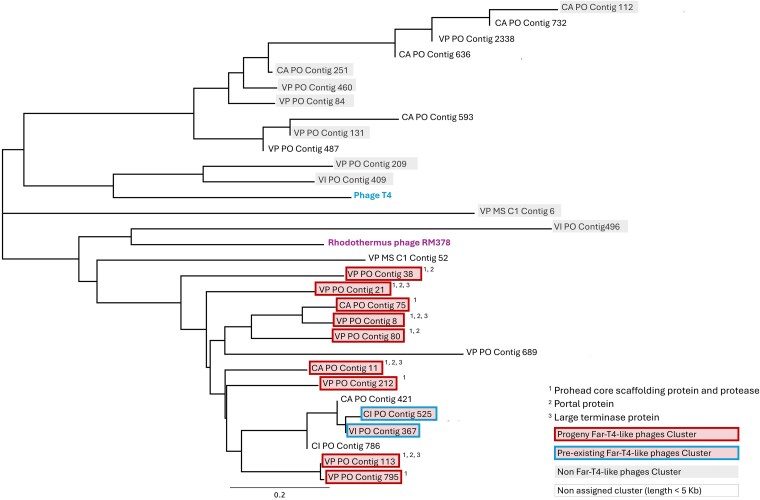
Phylogenetic tree of viral gp23 structural proteins as markers for T4-like phages. Maximum-likelihood phylogenetic tree reconstructed from gp23 protein sequences of viral genomes obtained by viral BONCAT-FACS. Highlighted boxes with a border indicate viruses affiliated with the Far-T4-like cluster shown in Fig. 3. Shaded boxes without a border represent viral contigs that cluster separately but encode a gp23-like structural protein. Contigs without a highlighted box correspond to viral BONCAT contigs shorter than 5 kb, excluded from the protein cluster network. Superscript symbols highlight viral genomes recovered in this study that share core proteins with Far-T4 phages.

Across all sorted cellular and viral samples, including both positive (i.e. newly produced viruses) and negative (pre-existing viruses) BONCAT fraction, we identified a total of 2192 viral contigs >1.5 Kb (Fig. 3, Table 1). Of these, 74% of contigs (*n* = 1630) contained two or more viral hits in the comparative analysis against the viral IMG/VR v4.1 database. Remarkably, nearly half of these viral contigs (*n* = 803) were detected in the BONCAT-positive viral fraction. This confirmed the successful recovery of BONCAT-labeled environmental viruses by FACS and demonstrated the feasibility of our overall workflow of viral BONCAT combined with FACS and single-virus sequencing [11, 28, 29, 41]. The population structure and diversity of BONCAT-positive (newly produced virions) and BONCAT-negative (pre-existing virions) viral contigs >5 Kb length (*n* = 1223) was analyzed by generating a protein sharing network using vConTACT2 [67] v.0.9.19 (Fig. 4). Similar viral genomes were recovered from both viral populations. However, different viral clusters within the network displayed large variations in the relative proportion of newly produced and pre-existing virions, which we defined here as the viral progeny ratio (i.e. normalized proportion of newly produced viral progeny vs. pre-existing viruses; see Supplementary Table 1 and Supplementary Data 1, Fig. 4). This comparative analysis identified population-specific differences in viral production and turnover rates among commonly recovered marine viruses. For example, viruses related to the uncultured pelagiphage vSAG 37-F6 [11, 62] (average amino acid similarity of ≈75%, Supplementary Data 2), one of the most abundant phages in the oceans [11, 62], were associated with a viral cluster that was dominated by newly produced, BONCAT-positive viruses (viral progeny ratio = 78.95%, *n* = 17; Fig. 4, Supplementary Table 1). Similarly, a second dominant viral cluster also stood out for being almost exclusively comprised of newly produced viruses (viral progeny ratio = 82.57%, *n* = 199; Fig. 4, Supplementary Table 1). This cluster contained several newly described uncultured Far T4-like phages from marine environments [63] and an isolated *Rhodothermus* phage RM378 [76] as the only reference genome. Phylogenetic analysis of the major capsid protein, Gp23, and other core proteins (large terminase, prohead, and portal proteins) from several of our recovered viral contigs in this cluster confirmed relatedness to these uncultured Far-T4 phages (Fig. 5; *n* = 15). The majority of the recovered Far-T4-like phage genomes (12 out of 15) came from the BONCAT-positive viral fraction, suggesting fast turnover of these viruses over the incubation period. Using the tool iPHoP [70], we screened for potential microbial hosts of the Far-T4-like phages. Several diverse host lineages were identified, with the largest fraction (113 out of 235, or 48%) belonging to the order Flavobacteriales (see Supplementary Data 3). Consistent with this finding, Flavobacteriales genome sequences were 15× more abundant in the BONCAT-active cell fraction (3% of the total bacterial reads) relative to the non-active sorted cells (0.2%) in the corresponding FACS-sorted microbial cell fractions (Supplementary Fig. 4; data available in Supplementary Data 4). The proportion of putative microbial hosts that were BONCAT-positive (active cells) in the seawater incubation, in tandem with the predominance of Far-T4-like phages in the viral progeny, is suggestive of ‘kill the winner’ dynamics, resulting in fast turnover of these viruses [77, 78]. While this current study focused on a single timepoint, future work using viral BONCAT-FACS for time-course experiments would enable longitudinal tracking of active and inactive cell and newly-produced virus populations, contributing to our understanding of host-virus dynamics. Finally, within Cluster 5 of Roseobacter-like viruses (Pacific Ocean sample), we detected a group of viral contigs (*n* = 8) present in all three fractions (newly produced viral progeny, metabolically active cells, and pre-existing free viruses). These contigs are related to members of a new family of globally distributed lytic roseophage-like viruses, Naomiviridae, (Viral cluster VC1099 [79]; >70% nucleotide identity) which has an unusual deoxythymidine to deoxyuridine substitution [80]. These viruses were recently recovered using single-particle genomics combined with semi-permeable capsules [79] (Supplementary Fig. 5, Supplementary Data 5 and 6).

**Table 1:** Assembled viral contigs assigned as viruses in each of the sorted populations.

**Sample**	**Population**	**Assembled contigs**	**Assembled 5 Kb**	**Viral assigned**	**Viral assigned 1.5 Kb**	**Viral assigned 5 Kb**	**Viral assigned 5 Kb, %**
Pacific Ocean	Viral progeny	21,141	1,606	1,584	960	543	33.8
	Pre-existing viruses	5,962	500	831	500	287	57.4
	Active cells	6,121	407	821	535	313	76.9
	Non-active cells	7,323	699	47	31	17	2.4
Mediterranean Sea	Viral progeny^a^	787	29	667	66	24	82.8
	Viral progeny^b^	679	69	476	77	39	56.5
	Active cells^a^	230	4	218	23	4	100.0

^a^6-h click.

^b^30-min click

### Analysis of viral contigs in the sorted active and inactive microbial cells and linking newly produced viruses and hosts

In addition to the information gained from direct sequencing of the sorted viral fraction, the analysis of the co-occurring microbial cells from the BONCAT-active and inactive fractions detected a greater proportion of viral contigs in the translationally active microbial cells than in the inactive ones in our seawater incubations. These viral contigs comprised ~77% of the total assembled contigs >5 Kb in the BONCAT-active microbial cell fraction, largely associated with the Caudoviricetes (Table 1, Fig. 3D). In contrast, the sorted inactive microbial fraction from the same sample (i.e. BONCAT-negative cells) contained only 2.4% viral contigs. These data suggest a higher infection rate and viral pressure among the translationally active members of the microbial community.

The taxonomic composition of the sorted BONCAT-active and inactive microbial cell fraction was also notably different. While the active microbial cell fraction sequenced from both field sites showed an abundance of *Pseudomonodota* (formerly Proteobacteria) representing ~72%–75% of the bacterial reads, the inactive cell fraction (coastal Pacific site) instead was largely associated with members of the Planctomycetaceae, representing ~28% of bacterial reads, with <0.1% in the active fraction (Supplementary Fig. 4, Supplementary Data 7 and 8). Among the BONCAT-sorted *Pseudomonodota*, members of the alpha- and betaproteobacteria were dominant and represented largely by the common marine lineages Pelagibacteriales (SAR 11) and Nitrosomonadales. Within the Nitrosomonadales, several taxa were affiliated with the methylotrophic clade OM43 [49], representing 2% and 17% of the assigned *Pseudomonodota* reads from the Pacific Ocean and the Mediterranean Sea, respectively (Supplementary Fig. 4, Supplementary Data 4 and 9). Described members of the OM43 clade are submicron-sized cells with streamlined genomes that are known for their ability to utilize C1 compounds and are common in coastal environments [49, 81].

A deeper characterization of OM43 diversity among the sequenced active and non-active cell fractions revealed the prevalence of a specific strain, *Methylophilales* sp. H5P1 [59], from the coastal Pacific Ocean site (Supplementary Fig. 6). Corresponding with the recovery of this bacterial strain, four of the assembled viral contigs from our paired sorted viral fraction were closely related to the methylophage group Melnitz, which has been previously shown to infect strain H5P1 [52] (Fig. 4, Supplementary Data 10). It is likely that the recovered BONCAT-labeled Melnitz-like phages originated from infections of *Methylophilales* spp., which represented one of the dominant bacterial lineages within the BONCAT-positive cell fraction. These data provide further evidence for the effectiveness of coupled cell- and viral BONCAT as a tool for assessing potential virus–host pairings.

Melnitz myophages have been reported from other temperate coastal and subtropical gyre environments, and our recovery of this lineage is consistent with earlier reports [52]. In a viral protein network, our four assembled methylophages co-occurred in a cluster with pelagiphages (HTVC008M, HTVC010P, HTVC011P, and HTVC019P) and cyanophages (Synechococcus phages) (Cluster 4, Fig. 4; more detailed in Supplementary Data 1), a feature that was also reported for previously isolated Melnitz phages [52]. Notably, these groups represent viral clades reported to have low production rates in the ocean [13, 82] and correspondingly, our BONCAT-estimated relative progeny ratio for the Melnitz-like phages group (Cluster 4) was 65.96% (*n* = 78; Fig. 4 and Supplementary Table 1), indicating lower turnover rates compared with other co-occurring phages like the Far-T4-like viral cluster.

### Recovery of eukaryotic viruses and parasitic virophages

Alongside the recovery of diverse bacteriophages, the viral BONCAT-positive fraction also recovered several eukaryotic viruses affiliated with nucleocytoplasmic large DNA viruses (Nucleocytoviricota or NCLDVs) [83]. A total of 12 viral contigs (>5 kb) associated with Phycodnaviruses, likely infecting algae, were recovered from the BONCAT-active virioplankton fraction from the coastal Pacific, with an additional four Megaviricetes viral contigs recovered in the Mediterranean Sea dataset. Within the Pacific Ocean samples, nine additional Phycodnavirus contigs were detected from the BONCAT-negative viral fractions. The detection of Phycodnaviruses also extended to the active cell fraction (*n* = 11), indicating active infection of microeukaryotes over the course of the incubation (Supplementary Fig. 7, Supplementary Table 2). Interestingly, alongside the Phycodnaviruses, genomes from two virophages (Lavidaviridae family) were also recovered in the same sorted BONCAT-positive viral fraction from the coastal Pacific (Supplementary Table 3). Virophages are parasitic phages that infect NCLDV viruses [84], and detection of an ongoing “superinfection” with viral BONCAT-FACS opens up new opportunities for studying infection dynamics in environmental samples for a broad diversity of bacteriophages and eukaryotic viruses alike.

## Discussion

In this study, we combined high-throughput BONCAT-FACS with advanced low-input viral genome sequencing technologies to investigate the fraction of newly produced viral progeny = within the total virioplankton, the smallest and most diverse biological entities in the ocean. Viral BONCAT-FACS provides a targeted method to differentiate and sort newly produced viruses and their microbial hosts based on translational activity, enabling genomic characterization of both BONCAT-labeled and -unlabeled components from the same sample. Although the sorting of viruses in this study was pooled, the BONCAT-FACS approach is fully compatible with single-cell technologies, holding the potential to resolve newly produced virions or infected cells at the level of single cell and single virus [85]. This represents a unique and complementary approach to community-wide methods in viral ecology such as metagenomics [86], transcriptomics [13–18, 20], stable isotope probing [21, 87], and radiotracer techniques [10], which lack the resolution to directly capture active infection of newly produced viruses at the single-cell and -virus level. The integration of activity-based, single-cell and -virus sorting with genomic analysis of the BONCAT-positive and BONCAT-negative fractions, yields information on often hidden dynamics within populations of viruses and their hosts, representing an important step toward developing a systems-level understanding of microbial interactions and activity within ecosystems. These datasets, combined with time series studies, can help refine ecological models of viral production, turnover, and their functions in regional and global biogeochemical cycles [88, 89].

The viral BONCAT method was initially developed as a fluorescence microscopy assay to visualize and quantify newly produced viruses in model microbial systems and seawater incubations [25, 40]. We have now adapted and optimized the BONCAT protocol for high throughput flow cytometry sorting and single-virus sequencing, enhancing fluorescence signal intensity to reliably detect, sort, and genomically characterize nanometer-scale viral particles and host microbial cells concurrently. The transition to flow cytometry required overcoming several technical limitations, as these submicron particles are at the theoretical limit of detection for most flow cytometers [11]. With this optimized method, we sorted both BONCAT-active and inactive microbial cells alongside viral particles from coastal seawater samples from the Mediterranean and Pacific and demonstrated its utility for resolving complex virus–host dynamics in the environment. Our recovery of OM43 infecting Melnitz-like phages from the same incubation as their previously described methylotrophic host, *Methylophilales* sp. H5P1 [52], provided confirmation of the ability of the viral BONCAT-FACS method to detect active infections and link viruses with their putative hosts within diverse environmental communities.

Across both the Mediterranean and the Pacific viral BONCAT datasets, notable variation in viral production across different lineages was observed. Specific SAR11-associated pelagiphages (e.g. vSAG 37-F6-like phages; recently classified as Pelagimarinivirus ubique within the new viral family Marinivirdae) and the recently described Far-T4-like phages, which we putatively linked to Flavobacteriales hosts, appeared to have high turnover, with large enrichments in the BONCAT-positive viral fraction. The recovery of BONCAT-positive phages belonging to the vSAG 37-F6 group was particularly noteworthy, as this uncultured pelagiphage group, originally characterized by single-virus genomics methods, was reported to be one of the most abundant and ubiquitous viruses in the marine virosphere [11, 28, 62, 90]. These viral BONCAT-FACS-generated data are consistent with previous reports from whole community viral metatranscriptomes from coastal environments that reported the high expression of vSAG 37-F6 transcripts relative to other pelagiphages, such as HTVC010P or HTVC011P, indicating a higher rate of host infection [13]. Our results expand upon these earlier findings, providing independent support for higher infection rates and turnover by the lytic vSAG 37-F6 group in coastal seawater among the co-occurring pelagiphages in our incubations. Given its ubiquity and activity in ocean ecosystems, this specific phage lineage is likely to be an important contributor to nutrient recycling through the viral shunt [91] and warrants further study (Fig. 4).

Our BONCAT-based estimate of lower viral production and turnover for other lineages related to cyanophages, methylophages, and the aforementioned HTVC010P and HTVC008M pelagiphages (e.g. Cluster 4, Fig. 4, Supplementary Table 1) was also consistent with their potential targeting of slower-growing bacterial hosts, as has been observed in earlier studies [13, 82]. Among viruses with lower viral production and turnover, we detected Rosephage-like viruses related to noncanonical marine DNA viruses of the novel family Naomiviridae, which use dU instead of dT, and their genomes could be sequenced only after producing canonical DNA copies with phi29 polymerase [80]. These viruses are totally undetectable using standard viral metagenomics like the one used in ‘Tara ocean’ expedition. Recent data point that these viruses could infect other abundant bacterioplankton, such as *Pelagibacter*, beyond the *Reseobacter* clade [79].

Other viral groups in the protein cluster network had even lower turnover rates, such as the phages in Cluster 1 likely infecting members of the Flavobacteriales, including *Cellulophaga* (Fig. 4, Supplementary Table 1 and Supplementary Data 1). This slow predicted turnover was distinct from the high progeny ratio calculated for the Flavobacteriales-associated Far T4-like cluster and, as observed among different pelagiphage lineages, highlights the variation in viral pressure among related host bacteria in coastal zones. Collectively, these lineage-specific patterns support ecological models such as ‘kill the winner’ [77, 92], where rapidly growing microbial host populations are impacted by elevated viral pressure and rapid phage production, while other phage lineages persist with comparatively lower production rates and turnover.

Beyond the dynamics detected among different phage lineages by viral BONCAT-FACS, the concurrent sorting and analysis of BONCAT-active and inactive microorganisms unexpectedly revealed substantial enrichment in viral contigs associated with the active cellular fraction (77%) relative to the inactive microbial cells (2.5%) in the same incubation (Table 1), suggesting that a high proportion of the translationally active planktonic bacterial community may be virocells [93–96]. The relationship between (presumed) viral infection and microbial activity is consistent with earlier reports demonstrating a positive correlation between total viral abundance and bulk bacterioplankton productivity [97]; however, the percentage of virocells in our BONCAT-FACS dataset is substantially higher than reported values in other FACS-enabled studies of bacterioplankton communities (~34% of cells) [12, 98]. This large discrepancy may be attributed to the fact that in these earlier studies, total cells were sorted rather than differentiated based on translational activity.

Because a positive BONCAT signal requires cellular uptake and incorporation of the methionine surrogate during protein synthesis, our findings suggest that microbial cells continue to actively take up nutrients just prior to, or during, viral infection. This interpretation aligns with prior stable isotope probing experiments in which *Synechococcus* infected by cyanophage continued to assimilate exogenous nitrogen and synthesize proteins throughout the infection cycle [99]. Should this pattern hold across systems, the ecophysiological interpretation of BONCAT activity may need to be broadened to reflect not just cellular anabolic activity, but also the presence of viral infection.

The microbe–virus interactions recovered in our viral BONCAT-FACS experiments were not limited to bacteria and their phages but also included several giant viruses known to infect microeukaryotes (NCLDV viruses belonging to the Nucleocytoviricota), highlighting the technique’s ability to capture a broad diversity of active microbes and lytic viruses. The simultaneous activity-based sorting of viruses and cells by FACS offers an important methodological advantage by directly capturing newly produced viral progeny across a broad size range, avoiding the size-selective bias that often excludes giant viruses from filtered submicron viral concentrates. The detection of potential virophage from the *Lavidaviridae* group within the active viral fraction indicates active production during the incubation (Supplementary Table 3). These virophages and their putative NCLDV hosts are widely distributed in ocean ecosystems [84, 100–105] and the nature of this super parasitism and its impact on host ecology remain areas of active study using diverse methods [19]. Future investigations that integrate the unique perspective of single-cell and virus-targeted BONCAT-FACS with synergistic viral ecology methods have the potential to expand our understanding of their ecology and the underlying interactions between microeukaryotes, viruses, and their parasites in nature.

While viral BONCAT-FACS yields unique insights, several methodological considerations warrant discussion. As with all incubation-based methods, BONCAT experiments may be subject to potential artifacts such as bottle effects, which can alter community composition and activity. Additionally, variability in HPG sensitivity and assimilation among microbial taxa may influence BONCAT results [106]. These factors should be independently evaluated when applying viral BONCAT to new environments. In the context of our study, the use of BONCAT in marine planktonic communities was previously assessed and found to yield results comparable with other methods. Prior investigations with coastal seawater, e.g. showed consistent results between BONCAT-based assays and independent measures of community productivity and viral production [25, 107]. Additionally, the low concentration of HPG used in our incubation experiments was below that reported to impact the growth of some cyanobacteria [106]. Despite the potential for perturbation, the application of viral BONCAT-FACS was able to successfully recover and genomically characterize newly produced marine viruses and diverse translationally active microbial hosts from coastal seawater.

The flow cytometry detection, sorting, and sequencing of viral particles with very small genomes (<10 kb) that are common for single-stranded DNA (ssDNA) and RNA viruses currently remain a challenge [28]. However, recent advances in spectral and imaging flow cytometry show promise [108, 109]. Complementing our results using standard FACS, we also applied viral BONCAT-FACS using spectral flow cytometry to test reproducibility of the method across different platforms using the same samples and BONCAT protocol described in the Materials and Methods section (Supplementary Fig. 8). The spectral flow cytometer enabled enhanced detection of BONCAT-positive viral populations and improved efficiency during bulk sorting with the fluorophore AF647-Picolyl-azide (Supplementary Fig. 8). A wide variety of fluorophores (both alkyne- and azide-based) are available for BONCAT, and we selected AF647-Picolyl-azide due to its strong quantum yields (ε 270.0 l mmol-1 cm-1). This dye offered key advantages over other fluorophores for detecting small biological particles like viruses and, importantly, also allowed for counterstaining with SYBR (a general nucleic acid stain) to detect total viruses. We note that the 647 signal can overlap with chl-a autofluorescence using standard FACS, potentially generating a bias with phototrophic microorganisms; however, the spectral flow cytometer can differentiate between the BONCAT fluorophore and chl-a autofluorescence, suggesting this may be an ideal instrument for experiments where chl-a containing microorganisms are abundant. In the context of our study, our FACS analysis of the negative control incubations (without HPG) for the coastal seawater samples showed a minimal number of autofluorescent cells (Fig. 2D and E, Supplementary Fig. 2E), and we did not observe an overrepresentation of cyanobacteria in the sequenced BONCAT-positive cell fraction, indicating that the potential bias from chl-a cells in our BONCAT sorts was minimal.

## Conclusions

The development and successful application of viral BONCAT-FACS represent an important advancement in viral ecology, complementing existing omics-based methods. This activity-based sorting and genomic-sequencing approach enables the differentiation of newly produced viruses and their actively infected microbial hosts from co-occurring inactive populations in natural communities. Our initial application of viral BONCAT-FACS yielded insights into diverse marine virus–host interactions in coastal ecosystems, with lineage-specific differences in turnover predicted for viruses infecting common members of the bacterioplankton, including Flavobacteriales, SAR11, OM43, Cellulophaga, and eukaryotic phototrophs. Furthermore, noncanonical marine DNA viruses belonging to Naomiviridae were detected actively infecting the bacterioplankton. The unexpected finding of what appears to be a high prevalence of virocells among translationally active members of microbial communities suggests a reconsideration of traditional interpretations of microbial activity may be needed and highlights new directions for exploring the impacts of viral infection and its influence on nutrient cycles. Looking forward, the application of viral BONCAT-FACS alongside other viral ecology methods holds promise for revealing previously hidden virus–host interactions across diverse ecosystems, from deep-ocean sediments to industrial bioreactors and animal and plant-associated microbiomes, expanding our systems-level understanding of microbial communities and their ecological and biogeochemical significance.

## Supplementary Material

ycag048_Supplementary_Material_Alvarez_accepted_March2026

ycag048_Fig_Supp_8

ycag048_Fig_Supp_1

ycag048_Fig_Supp_2

ycag048_Fig_Supp_3

ycag048_Fig_Supp_4

ycag048_Fig_Supp_5

ycag048_Fig_Supp_6

ycag048_Fig_Supp_7_new

ycag048_Supplementary_Data_1_10

## Data Availability

The raw reads from the active and non-active cell and viral progeny and pre-existing viruses are available on the SRA data bank under accession numbers SAMN53290052, SAMN53290053, SAMN53290054, SAMN53290055, SAMN53290056, SAMN53290057, SAMN53290058, SAMN53290059, and SAMN53290060 from the BioProject PRJNA1365689.

## Disclaimer

This report was prepared as an account of work sponsored by an agency of the US Government. Neither the US Government nor any agency thereof, nor any of their employees, makes any warranty, express or implied; assumes any legal liability or responsibility for the accuracy, completeness, or usefulness of any information, apparatus, product, or process disclosed; or represents that its use would not infringe privately owned rights. Reference herein to any specific commercial product, process, or service by trade name, trademark, manufacturer, or otherwise does not necessarily constitute or imply its endorsement, recommendation, or favoring by the US Government or any agency thereof. The views and opinions of authors expressed herein do not necessarily state or reflect those of the US Government or any agency thereof.
