# Supplementary material for: Illuminating the newly produced viruses within the virosphere with bioorthogonal noncanonical amino acid tagging and single-virus genomic sequencing technologies
Source: ISME Commun. 2026 Mar 6;6(1):ycag048. doi: 10.1093/ismeco/ycag048 (PMC13037479; doi:10.1093/ismeco/ycag048)
Supplement: ycag048_Supplementary_Material_Alvarez_accepted_March2026 [file ycag048_supplementary_material_alvarez_accepted_march2026.docx]

**Supplementary Material for**

**Illuminating the newly produced viruses within the virosphere with BONCAT and single virus genomic technologies**

Maria Alvarez-Sanchez^1,2^ and Francisco Martinez-Hernandez^1,3^, Aitana Llorenç Vicedo^1,2^, Marina Vila-Nistal^1^, Alon Philosof^3^, Aditi K. Narayanan^3^, Jamie C. Tijerina^4^, Oscar Fornas^5,6^, Manuel Martinez-Garcia*^1,2^, Victoria J Orphan*^3,7^

^1^Department of Physiology, Genetics, and Microbiology, University of Alicante, Carretera San Vicente del Raspeig, San Vicente del Raspeig, Alicante, 03690, Spain

^2^Multidisciplinary Institute for Environmental Studies (IMEM), University of Alicante, Carretera San Vicente del Raspeig, San Vicente del Raspeig, Alicante, 03690, Spain

^3^Division of Biology and Biological Engineering, California Institute of Technology, Pasadena, CA 91125, USA.

^4^Caltech Flow Cytometry Facility, California Institute of Technology, Pasadena, CA 91125, USA.

^5^Centre for Genomic Regulation (CRG), The Barcelona Institute for Science and Technology (BIST), Carrer del Doctor Aiguader, 88, PRBB Building, Barcelona, 08003, Spain

^6^Universitat Pompeu Fabra (UPF), Carrer del Doctor Aiguader, 88, PRBB Building, Barcelona, 08003, Spain

^7^Division of Geological and Planetary Sciences, California Institute of Technology, Pasadena, CA 91125, USA.

***Correspondence to:** [**m.martinez@ua.es**](mailto:m.martinez@ua.es) **and** [**vorphan@caltech.edu**](mailto:vorphan@caltech.edu)

Underlined authors contributed equally to this work

The authors declare no competing interests.

**This supplementary file contains a Method section, references, 8 figures and 3 tables. A separate Excel file titled “Supplementary_Data_1–10.xlsx” includes ten supplementary datasets described in the section “Supplementary Data Legends” below.**

**METHODS**

**Sample collection and processing**

Two independent experiments were conducted using seawater samples from the California Pacific coast and Mediterranean Sea. Pacific coast seawater sample (0.50 L) was collected from Corona del Mar at the Kerckhoff Marine Laboratory, owned and operated by the California Institute of Technology (California, USA, 33° 35′ 47.6″ N, 117° 52′ 48.9″ W) on April 29^th^, 2023. Mediterranean surface seawater sample (4.00 L) was collected from Cape the Huertas (Alicante coast, Spain, 38° 21′ 14.3″ N, 0° 25′ 36.6″ W) on November 9^th^, 2023. In both cases, seawater was transported to the laboratory as quickly as possible, within a time frame of less than 2 hours. A total volume of 0.25 L (Pacific coast) and 2.00 L (Mediterranean Sea) were incubated with 10 µM of l-Homopropargylglycine (HPG; amino acid analog of methionine; ref: CLK-1067-100, Jena Bioscience) in Erlenmeyer flasks for 5 days at 18ºC and under light:dark photoperiods of 12h:12h (Mediterranean coast) and 14h:10h (Pacific coast), capturing the average temperature and photoperiod of the sampling site. In parallel, for each sampling point, a control incubation using the same volume (0.25 and 2.00 L) of seawater incubated without HPG under the same conditions, was run to discriminate between the active and non-active portions of the microbial community by FACS after fluorescent labeling using click chemistry. From the Mediterranean Sea sample, 0.25 L were used for the experiment using a 30 min click reaction, while the rest of the volume (1.75 L) were used for an extended 6 h click reaction. From the Pacific coast seawater sample, the entire seawater volume was fluorescently labeled using the extended 6 h click reaction protocol. After a 5 day incubation, the seawater samples (with and without HPG) was concentrated to an approximate volume of 15 mL using the tangential flow filtration system Vivaflow 30,000 MWCO PES cassettes (Vivaflow 200, Sartorius), followed by further concentration using Amicon Ultra Centrifugal filters, 100 kDa (Amicon Ultra, Merck Millipore) with centrifugation (2,500 x g, 3 min; Microcentrifuge Labnet™ Spectrafuge™ 24D, Labnet) to a final volume of 208 µl. Samples were then washed with 5 ml of nanoparticle-free 0.02-μm-filtered 1× PBS, pH 7.4 and concentrated by centrifugation (2,500 x *g*, 3 min). This washing process was repeated 3 times. To reduce the viral decay and cell activity, samples were maintained at 4ºC throughout the concentration and washing process.

**BONCAT-click chemistry fluorescent labeling of translationally active cells and viral progeny**

Standard click reaction for fluorescently labeling incorporated HPG in active cells [1,2] and viral progeny [3] was optimized and performed in solution, without fixation, to allow the discrimination, sorting, and genome recovery of newly produced viral progeny and active cells using a combination of FACS and single-cell and -virus sequencing technologies. To minimize oxygen exposure during the click reaction, the reaction was conducted in a PCR tube (250 µl) as follows: 208 µl of sample volume and 42 µl of click reaction mix (see below). Blanks for the BONCAT reaction used 208 µl of 1X sterile PBS mixed with the same volume of click reaction mix. The click mix was made in two steps which included a dye premix (0.5 mM CuSO_4_, 2.5 mM THPTA and the 0.1 µM dye, final concentrations) and the reaction buffer (25 mM Aminoguanidine, 25 mM Sodium Ascorbate final concentrations, and 1X PBS). Working solutions for 20 mM CuSO_4_ (dissolved in 0.02 µm filtered MilliQ water), 100 mM Aminoguanidine, and 100 mM Sodium Ascorbate (both dissolved in 0.02 µm filtered 1X PBS) were prepared freshly just before starting the click reaction. The 50 mM THPTA, the 10 µM azide-modified dye (AF647-Picolyl-Azide, Jena Bioscience) stock solutions were previously diluted in 0.02 µm filtered MilliQ water and DMSO, respectively, and frozen at -20 ºC until use. To remove the dissolved oxygen, immediately before the preparation of the click mix, all the reagents and the 0.02 µm filtered 1X PBS, were individually bubbled with Argon gas for 15 seconds. To prepare 250 µl of click mix, we first combined 12.5 µl of 50 mM THPTA, 6.25 µl of 20 mM CuSO_4_, and 2.5 µl of 10 µM dye, followed by gently gassing the headspace of the 250 µl PCR tube with Argon and incubating for 3 min in the dark. This was followed by adding 62.5 µl of 100 mM Aminoguanidine, 62.5 µl of 100 mM Sodium Ascorbate, and 104 µl of 1X sterile PBS to this mixture and incubating for an additional 3 min in the dark. 42 µl of the final click mix was then added to fresh PCR tubes containing 208 µl of sample concentrate (also previously gassed with Argon). To avoid bubble generation, the click mix was carefully added along the walls of the PCR tube. The air space above the sample was then bubbled with argon, covered before sealing with a small piece of parafilm, and the PCR tube was gently inverted a couple of times to carefully mix the sample and the click mix. The samples with the click mix were incubated for 30 minutes (Mediterranean samples) or 6 hours (for the Pacific coast and the Mediterranean Sea samples) at room temperature in the dark. The 30 min click reaction time corresponds to the standard BONCAT protocol for translationally active cells[2,4] while the 6 h reaction was tested in our study and found to enhance the BONCAT fluorescent signal for viruses and improve their discrimination during FACS. Microbial BONCAT-positive cells were also clearly distinguished with the 6 hour protocol. After the click reaction, samples were concentrated and washed with 0.02-μm-filtered 1X PBS using Amicons (100 KDa, Millipore) to remove any unbound dye as follows: The Mediterranean Sea samples were concentrated (1x), followed by a wash step (3x) using 5 ml 0.02-μm-filtered 1× PBS in 100 KDa 15 ml Amicons (2,500 x g, 3 min), and finally resuspended in 0.02-μm-filtered 1× PBS, 500 µl. The Pacific coast samples were concentrated and washed (1x) with 500 µl of 0.02-μm-filtered 1X PBS using 0.5 mL Amicons (100 KDa, Millipore) by centrifugation at 7,500 x g for 3 min and then resuspended in 0.02-μm-filtered 1× PBS, 500 µl. The Pacific coast sample (500 μl final volume) was additionally counterstained with 2.5 μl 1,000 x SYBR^TM^ Gold, incubated for 20 min in the dark, and then washed 3 times with 500 µl of 0.02-μm-filtered 1X PBS in 0.5 mL 100 KDa Amicons (7,500 x g, 3 min). All click stained samples were immediately analyzed and sorted by FACS the same day or within 12 h of completing the reaction. In all cases, samples were maintained at 4ºC in the dark until sorting.

**Fluorescent microscopy protocol for click reaction with different incubation times**

To optimize the click-chemistry protocol for improved detection by flow cytometry, 500 mL of Pacific Ocean seawater was incubated with 10 μM HPG as described above. It is important to note that, although this procedure followed the same workflow, the samples used here originated from an independent experimental replicate. Following HPG incubation, the sample was concentrated and washed with 0.02-μm–filtered 1× PBS using 100 kDa Amicon centrifugal filters (7,500 × g, 3 min). The concentrate was then divided into two equal volumes, and the click reaction was performed on both aliquots, differing only in reaction time (30 min versus 6 h). In parallel, 500 mL of seawater without HPG was processed identically as a negative control.

After the click reaction, each sample was concentrated and washed once with 500 μL of 0.02-μm–filtered 1× PBS, resuspended in 250 μL of 0.02-μm–filtered 1× PBS, and counterstained with 1.25 μL of 1,000× SYBR Gold in the dark for 20 min at room temperature. The samples were then concentrated and washed three additional times with 0.02-μm–filtered 1× PBS and resuspended in a final volume of 500 μL of filtered 1× PBS.

Half of each sample (250 μL) was analyzed by flow cytometry to assess differences in VLP detection, while the remaining 250 μL was filtered onto 0.02-μm Anodisc filters (Whatman) using a filtration column. Filters were air-dried in the dark and mounted with Citifluor AF1 antifade mounting medium (Electron Microscopy Sciences). Samples were visualized with an Olympus BX51TRF epifluorescence microscope equipped with a Plan Apo 100× (NA 1.4) objective and an X-Cite 120Q excitation light source. Filter sets used were Chroma FITC/Alexa Fluor 488 (Excitation: 480/40 nm, Emission: 535/50 nm) for SYBR Gold, and Cy5 (Excitation: 647/30 nm, Emission: 670/30 nm) for AF647. Increasing the click reaction incubation time from 30 minutes to 6 hours improved the fluorescence-to-noise ratio in microscopy imaging (Fig. 2A, B).

**Fluorescence-Activated Viral and Cell Sorting (FACS)**

FACS analysis and sorting of Pacific coast and Mediterranean samples were conducted in a BD FACSAria^TM^ Fusion (Becton Dickinson, San Jose, CA) at the Caltech Flow Cytometry and Cell Sorting Facility (Pasadena, CA, USA) and Central Service for Experimental Research (SCSIE) from the University of Valencia (Valencia, Spain), respectively. Strict DNA decontamination of instruments, reagents, and materials were performed as described in single cell genomic protocols by Rinke and collaborators [5,6] and further studies [7–10]. In brief, autoclaved sheath fluid was prepared in house with combusted NaCl, sterilized and overnight UV-irradiated. Sheath fluid tank was autoclaved, UV-irradiated, and also decontaminated by washing with 10% bleach, for at least 10 minutes (up to 30 minutes) and rinsed with sterile UV-irradiated (16 h) autoclaved MilliQ water. Then, sheath fluid tank was filled with sterile sheath fluid free of DNA. All this process was carried out on a sterile environment within a PCR hood.

The sorter was setup to detect the red AF647-Pycolyl-Azide (Abs/Em=648/671 nm) fluorescence from BONCAT positive virus and bacteria using the 640 nm red laser. The green fluorescence signal from total (active and non-active) bacteria and DNA-containing viral populations labeled with SYBR^TM^ Gold (Abs/Em=495/537 nm) was excited using the 488 nm blue laser. Both fluorescent channels were represented versus the side scatter height (SSC-H). To delimitate the flow cytometer regions for the different populations, 1X PBS and incubated seawater without HPG were identically processed as samples stained with SYBR^TM^ Gold and BONCAT-click (Supplementary Fig 2A-C*)*. Samples were also additionally filtered by 0.2 μm to better discriminate viral and cell communities (Fig. 2D). After analyzing samples and defining populations compared to negative controls, seawater sample with HPG double staining (BONCAT/SYBR) were first observed in the SYBR channel vs. SSC-H (Supplementary Fig. 2 D). Gates were adjusted in SYBR channel for total cells and viral-like particles (VLPs), and each population was visualized in BONCAT channel vs. SSC-H plots. Subsequently, seawater samples without HPG and double staining were run as negative controls, visualizing total cell/VLPs populations in SYBR vs. SSC-H followed by visualized echa os these populations in the BONCAT channel vs. SSC-H (Supplementary Fig 2E). By comparing BONCAT fluorescence of SYBR+ cells and VLPs SYBR + between HPG and no-HPG samples, BONCAT+ thresholds were set, defining sorting gates for BONCAT+/BONCAT- and enabling sorting of active cells, inactive cells, viral progeny, and pre-existing viruses (Supplementary Fig. 2 D-E).

In the cause of Mediterranean samples, the active bacterial and viral progeny populations were sorted based on side scatter height (SSC-H) and APC (BONCAT+) fluorescence intensity, using the same negative controls to defining populations (PBS + BONCAT, seawater without HPG + BONCAT and filtered seawater HPG sample by 0.22 μm) as describe above (Fig 2). For the Pacific samples, bacterial and viral populations were counterstained with SYBR-Gold as described above and sorted based on the double threshold from FITC (SYBR-Gold) and APC fluorescence (BONCAT) (Figure 2D; Supplementary Figure 2D-E). All sorting experiments were carried out setting the flow cytometry sorter at the single cell mode, which employ the most stringent sorting purity conditions.

Up to five pool replicates of BONCAT-positive populations from Mediterranean samples were sorted in 96-well plates (1,000 and 5,000 viruses from viral progeny; 200 active cells) using a flow rate 40-800 ev/sec. For the Pacific samples, each BONCAT-positive and BONCAT-negative population were sorted into a single pool in 16 h UV irradiated 1.5 ml tubes (100,000 viruses from viral progeny, 50,000 pre-existing viruses and 25,000 active and non-active bacteria) and divided in wells from 96-well plate with 5,000 viruses or cells each (20 wells for viral progeny, 10 wells for non-active viruses and 5 wells for active and non-active bacteria). Furthermore, 10,000 BONCAT-positive viruses from Pacific Coast were sorted into a 1.5 mL Eppendorf tube for direct visualization by fluorescence microscopy using an Olympus BX51TRF epifluorescence microscope equipped with a Plan Apo 100× (NA 1.4) objective and an X-Cite 120Q excitation light source. The same Chroma filter sets described previously were used to identify SYBR Gold and BONCAT (AF647+) overlap signals (Figure 2C). During viral sorting, we used a FACS set up (e.g. flow rate) following the previously described protocol used for single virus FACS and genomic sequencing [10] to ensure efficient sorting of nanoparticles.

The sheath fluid signal detected in the APC channel as electronic noise was also sorted in the same 96-well sterile plates or new sterile 1.5 ml tubes and used as a negative control for multiple displacement amplification (MDA) and sequencing. Sorted cells and viruses were stored at -80 °C until whole-genome amplification.

**Whole-genome amplification of sorted bacterial and viral populations**

The DNA of the viral and cell pools was amplified by MDA using the EquiPhi29 polymerase (ref: A39392; Thermo Fischer scientific)[8], as described in Garcia-Heredia, et al., 2020 [11]. Briefly, after a thermal and chemical lysis of the viral capsid, the MDA master mix was added containing 0.26 U of Equiphi29 DNA polymerase (Thermo Fischer scientific), 1X EquiPhi29 reaction buffer (Thermo Fischer scientific), 0.8 µl of 0.04 mM Heptamers (IDT), 10 mM of DTT (Sigma), 0.4 mM of dNTPs (New England Biolab), 0.002 µl of SYTO 9 (Invitrogen) and 5.44 µl of sterile ultraviolet 16 h-treated MilliQ water. During the preparation, MDA master mix, except SYTO 9, and the DLB (0.4 M KOH, 10 mM EDTA and 100 mM dithiothreitol) and Stop solution (pH=4, Qiagen) used for viral/cell lysis were UV-light irradiated as described in Rinke et al., 2014 [5]. The final MDA reaction volume was scaled upon the volume of sorted samples. As MDA positive controls, we added 1 ng/µl and 10 ng/µl genomic DNA for the *E.coli* lambda phage, and 16 h-UV-irradiated 1X TE buffer as a negative control. The real-time monitored MDA reaction was carried out at 45ºC, and the reaction was stopped at 75ºC for 10 minutes once high fluorescence values, indicative of high DNA content, were reached [10]. Reaction times varied between approximately 3.5 and 5.5 hours for Pacific samples and up to 16 hours for Mediterranean samples. This variation was due to differences in the number of events sorted per well. All Pacific sample wells successfully amplified, while 77% of Mediterranean sample wells yielded successful amplification. None of the wells containing sorted sheath fluid showed successful amplification, confirming the effectiveness of the DNA decontamination and cleaning procedures for FACS. Plates with MDA amplified DNA were stored at –80ºC until further use.

**Sequencing and assembly of amplified genomes from viral and bacterial populations**

All the MDA products from the Pacific Coast samples were sequenced (100,000 BONCAT-positive viruses, 50,000 BONCAT-negative viruses, and 25,000 active and non-active bacteria). A subset of 5 pools was selected from the Mediterranean Sea for sequencing; 3 from the 30 min click reaction (1 x 200 active cells and 1 x 1,000 and 1 x 5,000 BONCAT-positive viruses) and 2 from 6h click reaction (1 x 200 and 1 x 5,000 active cells and BONCAT-positive viruses respectively). Libraries were prepared using the Illumina DNA Prep (M) kit and adapters from IDT for Illumina DNA/RNA UD, following the manufacturer’s protocol. Illumina paired-end sequencing was performed with NovaSeq 6000 (150 x 2 PE; approximately 3 Gb per sample) by Macrogen (South Korea). The obtained sequence reads were quality-trimmed using Trimmomatic 0.39 [12] with the following settings: -phred33 LEADING:3 TRAILING:3 SLIDINGWINDOW:4:30 MINLEN:50. Quality-filtered reads were assembled using Spades v3.15.5 [13] with the following settings: --sc mode, -k 21,33,55,77,99,127.

**Taxonomic analysis of bacterial reads**

Trimmed and unassembled reads derived from both active and non-active bacterial samples were taxonomically analyzed using Kaiju [14], and compared against the NCBI BLAST database (nr_euk) which includes data from bacteria, archaea, eukaryotes and viruses, with the following parameters: -E 0.00001, -a greedy. Taxonomic profiles for the samples were generated and analyzed with krona [15]. For the Pacific coast samples, only a 6 h click reaction was performed, as this longer incubation was optimized to enhance BONCAT fluorescence and improve detection of BONCAT-positive viral populations. From this incubation, both the viral and bacterial populations were clearly discriminated by FACS and subsequently sorted for taxonomic analysis. In contrast, the Mediterranean experiment included both a 30 min (standard BONCAT bacterial protocol) and 6 h click reactions. BONCAT-positive microbial populations were identified and sorted from both reaction times. All microbial datasets were analyzed using kaiju; however, the 30 min incubation yielded a higher proportion of taxonomically classified reads compared to the 6 h sample. As such, the downstream bacterial analyses shown correspond to the 30 min sample for the Mediterranean experiment and to the 6 h sample for the Pacific experiment.

**Viral identification and bioinformatic genome analysis**

For detecting and classifying viral contigs, after assembly, a combination of Virsorter2.0 (v2.2.3) [16] and CheckV (v0.9.0) [17] (following the viral sequence identification SOP V.3 from the Sullivan Lab, [**dx.doi.org/10.17504/protocols.io.bwm5pc86**](https://dx.doi.org/10.17504/protocols.io.bwm5pc86)), and geNomad (v1.7.0; default parameters) [18] was used. In addition, to corroborate the viral origin of the detected viral contigs, and better characterize some specific viral groups, the ORFs from all contigs ≥ 1,500 bp were predicted using Prodigal v2.6.3 [19] and compared against the high confidence viral proteins from the IMG/VR v4 [20] with Protein-Protein BLAST 2.8.1+ using the following settings: *-outfmt "6 qseqid sseqid pident length mismatch gapopen qstart qend sstart send evalue bitscore qlen slen positive ppos", -evalue 0.00001*. We considered as *bona fide* viruses all these genomes detected by the viral identification SOP, also by geNomad and sharing more than 2 proteins (thresholds of 80% similarity and a coverage of 90%) with viral contigs from IMG/VR.
To search OM43 phages within our viral contigs we employed as reference the isolated OM43 phages available from NCBI, the phage Venkman EXVC282S [21], the Methylophilales phage MEP301 [22], the Methylophilales phages Melnitz (EXVC039-EXVC044M) [23], the Methylophilales phages MEP401-MEP402 and their related 99 metagenomic viral genomes found in viral databases [24].

The reference genome of the vSAG 37-F6 [10,25], considered one of the most abundant and widespread marine viruses, was also used to search similar viruses within that population. This lineage has been recently classified as Pelagimarinivirus ubique within the new viral family Marinivirdae, Vila-Nistal et al., 2025 [45]. Following the same protocol described above, their proteins were blastp compared with all the viral-BONCAT genomes, finding 2 viral contigs sharing over 16 viral proteins, with an average amino acid similarity of approximately 74-75%.

To polish the taxonomic annotation, those contigs > 5,000 bp detected with geNomad from all samples as “giant” viruses and virophages (Phylum Nucleocytoviricota and Preplasmiviricota, respectively) were manually verified with the taxonomic information from the IMG/VR metadata.

**Viral protein network**

To build a network clustering similar viruses based on protein sharing we employed vConTACT2 (v0.9.19) [26] through the DOE Systems Biology Knowledgebase (KBase, [http://kbase.us](http://kbase.us/)) [27] using as reference database the “Prokaryotic Viral RefSeq 201”, protein cluster method “MCL” and viral cluster method “ClusterONE”. The viral network was visualized using Cytoscape v3.7.1 [28].

As an indicator of the viral turnover rate of each viral population we estimated the relative viral progeny ratio as the percentage of viral BONCAT positive viruses over the total viruses (active and inactive) from a cluster (e.g. Fig. 4 and Supplementary Table 1). As only inactive viruses from the Pacific Ocean were sorted and analyzed; calculations of viral progeny ratio only used datasets from the Pacific Ocean viral genomes associated with the BONCAT positive and negative-sorted free virions. To account for differences in sequencing depth caused by the initial sorting strategy (which included 100,000 viral progeny viruses but only 50,000 inactive viruses) we normalized the data using the formula:

1. Viral progeny ratio (%) = 100 x number of active viruses (viral progeny)/ (number of active + 2 x number of inactive viruses).

This correction reflects that approximately twice as many viral genomes were retrieved from viral progeny samples (n=543) as from inactive ones (287), thereby avoiding overestimation of active viruses in the dataset (detailed calculations of the viral progeny ratio for each cluster, along with counts of active and inactive viruses, are provided in Supplementary Data 1).

**Far-T4 phages identification**

For the Far-T4 phages identification we used an approximation like that used by Roux, S., *et al*., 2015 [29], screening all the viral BONCAT contigs > 1,500 bp for the presence of the T4 major capsid protein Gp23. All Gp23-like protein (protein to protein blastp bit score > 50 and e-value < 1x10^-10^) were selected to build a phylogenetic tree. For it, all the Gp23-like proteins were aligned using MAFFT v7.310 (--auto) [30], columns with gaps in more than 80% of the sequences were removed from the alignment using trimAI v1.4 (-gt 0.2) [31] and then the tree was constructed using Geneious bioinformatic software [32]. In parallel, all the proteins from our viral contigs were blastp-compared against the proteins of *Rhodothermus RM378 phage* (as the only isolated representative of the Far-T4 phages group) to check the presence of core proteins.

**Host assignment of the Far-T4 phage cluster**

To computationally identify the hosts of the viruses contained the Far-T4 phage cluster, we run the iPHoP machine learning (v1.3.3) [33] using the host database “*iPHoP_db_Aug23_rw*” based on GTDB r214 and extended collection of MAGs. Due to that application did not show significant results (probably due to the completeness percentage of our sequences), we opted for an indirect approach; we compared all the proteins of the viruses from the Far-T4 phages cluster with those of the high confident IMG/VR v4 database [20] using blastp. To be conservative, we selected those IMG/VR genomes sharing at least the 80% of the proteins of our contigs (not less than 5 proteins) with > 60% of amino acid similarity and >95% amino acid coverage of the alignment. Then iPHoP was applied to that IMG/VR contigs as described above.

**Bacterial read fragment recruitment against OM43 genomes**

To estimate the abundance of the OM43 clade in active and non-active bacterial fraction from Pacific and Mediterranean seawater, we performed a read recruitment plot analysis [34] using 13 publicly available genomes at NCBI derived from isolates OM43 HTCC2181 [35], KB13 [36], MBRSH7 [37], H5P1 [23] and LSUCC0622, LSUCC0268, LSUCC0389, LSUCC0401, LSUCC0536, LSUCC0568, LSUCC0603, LSUCC0665, LSUCC0717 [38]. Trimmed reads derived from bacterial samples were subjected to BLASTn analysis (BLAST 2.8.1+) against concatenated OM43 genomes using the following settings: blastn -outfmt "6 qseqid sseqid pident length mismatch gapopen qstart qend sstart send evalue bitscore qlen slen ", -evalue 0.00001, and applying a threshold of 70% similarity and a coverage of 70%. The BLASTn output was filtered using the BestHit Script available by enveomics tools [32] and recruitment plots were carried out using enve.recplot 2 (R stadistic pluging).

**Analysis of *bona fide* viral BONCAT contigs related to non-canonical marine Roseophages**﻿

To investigate whether non-canonical marine viruses with unusual deoxythymidine-to-deoxyuridine (dU) substitution in their DNA can be detected with our viral BONCAT approach, we compared the 1,630 *bona fide* viral BONCAT contigs recovered from all Pacific Ocean and Mediterranean Sea fractions against representative genomes of these phages. These reference sequences included two cultured roseophages, DSS3_VP1 and DSS3_PM1 (GenBank accession numbers MN602266 and MN602267) [39], and a recently described uncultivated virus obtained by microfluidic single-cell genomics [40], (Viral cluster VC1099SCGC;_Capsule1_10222112 (GenBank BioProject PRJNA1227513). All reference genomes were concatenated into a single FASTA file and used as database in a nucleotide similarity search BLASTn analysis (BLAST 2.8.1+; parameters: -outfmt "6 qseqid sseqid pident length mismatch gapopen qstart qend sstart send evalue bitscore qlen slen" -evalue 1e-5) against the concatenated *bona fide* viral BONCAT contigs.

Thirteen viral BONCAT contigs showed hits to SCGC_Capsule1_10222112 with nucleotide identity between 72–85% over a single viral genome (Supplementary Data 5).﻿ These 13 contigs were derived from different FACS fractions: 5 from active cells, 5 from pre-existing viruses, and 3 from viral progeny, all from the Pacific Ocean dataset. Predicted proteins from these 13 viral BONCAT contigs and from SCGC_Capsule1_10222112 were generated with Prodigal [19] and compared using Protein-Protein BLAST 2.8.1+ (parameters: -outfmt "6 qseqid sseqid pident length mismatch gapopen qstart qend sstart send evalue bitscore qlen slen positive ppos" -evalue 1e-5). This analysis recovered 53 viral BONCAT proteins with matches to SCGC_Capsule1_10222112 proteins, showing >80% positive similarity and ≥90% alignment coverage (Supplementary Data 6).

Of the 13 viral BONCAT contigs identified as putative non-canonical Roseophages, 8 were placed in network cluster 5 together with the reference genomes Roseobacter virus SIO1, Celeribacter phage P12053L, Lentibacter virus vB_LenP_ICBM1, Lentibacter virus vB_and LenP_ICBM2 (Supplementary Data 1), consistent with previous phylogenetic analyses of these isolates [39]. The remaining 5 contigs were not represented in the network because their length was <5,000 bp, and only viral BONCAT contigs ≥5,000 bp were included in the network analysis. Sequence alignment similarity of three of the viral BONCAT contigs with the longest blastn lengths (2050, 2050 and 2124 bp; Supplementary Data 6) was performed against the Naomiviridae reference genome VC1099 using CLUSTAL W (Supplementary Fig. 5) [41]. Open reading frames (ORFs) were predicted using Genmark [42], and annotation was done using BLAST conserved domain search and BLASp.

**BONCAT in *Escherichia coli* culture infected with phage T7**

BONCAT incubations were carried out with *E. coli* K12 (kindly provided by Dr. Francis Mojica, University of Alicante) and bacteriophage T7 (kindly provided by Dr. Mark van Raaij, Centro Nacional de Biotecnología-CSIC, Spain). Bacterial growth and infection procedures were based on the protocol described by Pasulka et al. (2018)[3] with several modifications to adapt them to our experimental setup. An overnight culture of *E. coli* K12 was prepared in Luria-Bertani (LB) medium and incubated at 37 °C with shaking. The following day, 1.5 mL of this culture was used to inoculate 150 mL of M9 minimal medium and grown at 37 °C until reaching an optical density at 600 nm (OD_600_) of 0.6. The M9 medium used in these experiments was prepared by combining 30 mL of 5× M9 salts (Sigma, ref. M6030-1KG; containing 2.5 g NaCl, 5 g NH₄Cl, 64 g Na₂HPO₄ x 7H₂O, and 15 g KH₂PO₄ per 200 mL solution), 300 µL of 1 M MgSO₄ (final concentration 2 mM), 50 µL of 1 M CaCl₂ (final concentration 0.3 mM), 1.5 mL of 20% glucose (final 0.2%), 3 mL of 0.5% thiamine (final 0.01%) and 114.87 mL of deionized water. To label newly synthesized proteins, l‑homopropargylglycine (HPG; Jena Bioscience) was added to exponentially growing *E. coli* cultures at OD_600_ of 0.3 to a final concentration of 100 µM. Cultures were allowed to grow for approximately until OD600 = 0.6 before infection. At this point, T7 phage suspensions (6.4 × 10^10^ VLPs/ml) were added to both HPG‑treated and control cultures at a multiplicity of infection (MOI) of 0.3. Cultures were then incubated under agitation at 37 °C until complete cell lysis was observed (overnight). Following lysis, 10 mL of each culture were centrifuged at 2,935 × g for 15 min at 4 °C to remove cell debris. The resulting supernatants were filtered through 0.22 µm pore filters, and 3 mL of each filtrate were concentrated using 15 mL Amicon Ultra centrifugal filters (Millipore). The concentrates were washed three times with 5 mL phosphate‑buffered saline (PBS) by centrifugation at 2,500 × g for 3 min. The final concentrates were recovered in 500 µL of sterile buffer PBS 1X. From each preparation (samples and blanks) 208 µL were used for the subsequent click‑labelling reaction.

Click reaction was performed following the above described BONCAT protocol with a 30 min reaction time. After the reaction, samples were washed three times with 5 mL of 1x PBS buffer (centrifugation at 2500 x g for 3 min each) and resuspended in 1 ml of 1x PBS buffer.

For confocal microscopy analysis, 50 μl of the labeled sample was mixed with 1.25 μl of SYBR Gold (1:1000 dilution; final concentration 0.25X) and incubated in the dark for 20 min. Labeled samples were placed as droplets on Superfrost Plus glass slides (Thermo Scientific), air-dried, and mounted using Citifluor AF1 mounting medium (Electron Microscopy Sciences). Samples were visualized using a Zeiss LSM 800 Confocal Scanning Microscope (Carl Zeiss Microscopy, Germany) equipped with an Alpha Plan-Apochromat 100x/1.46 Oil DIC (UV) M27. Fluorescence signals were detected using two independent channels. The BONCAT signal was captured using a 640 nm excitation laser with emission detected between 645 and 700 nm (60% intensity laser, detector gain = 650 V), while SYBR fluorescence was excited with a 488 nm laser and collected between 450 and 700 nm (100% intensity laser, detector gain = 650 V). To prevent spectral overlap between channels, images were acquired using a sequential scan mode, recording each fluorophore separately from the same field of view. Two images (only BONCAT, only SYBR) were then merged digitally. Merged images were analyzed to evaluate potential overlap between T7 phage particles detected in the BONCAT and SYBR channels. T7 without HPG was included as a negative control to establish the background signal threshold for the BONCAT channel (Supplementary Fig. 3A).

For flow cytometry, samples were fixed with 0.5% glutaraldehyde for 20 min a rapidly frozen using nitrogen. A blank control containing only PBS buffer was prepared under same fixation conditions. Staining for cytometry analysis was performed by adding 5 µL of 50X SYBR Gold stock solution to 500 µL of fixed sample and incubating for 15 min at 80ºC, as described in Corina et al., 2010 [43].

Flow cytometry and cell sorting were performed using a BD FACSAria Fusion (Becton Dickinson, San Jose, CA, USA) at the Scientific and Technological Centers of the Autonomous University of Barcelona (Barcelona, Spain). Prior to analysis, all instruments, reagents, and materials were subjected to strict DNA decontamination as described above for single-cell genomic technologies.

The sorter was configured to detect red fluorescence from BONCAT labeled T7 phages carrying the AF647-Picolyl-azide fluorophore (excitation/emission = 648/671 nm) using a 640 nm red laser. Total viral particles were simultaneously detected based on green SYBR Gold fluorescence (excitation/emission = 495/537 nm) using a 488 nm blue laser.

To define the regions corresponding to BONCAT positive T7 particles, fluorescence plots of BONCAT intensity versus side scatter height (SSC-H) were compared with negative controls (PBS and T7 samples without HPG). Based on these comparisons, the gating strategy was refined by plotting BONCAT (signal on x axis) against SYBR Gold (signal on y axis), enabling identification of the SYBR+BONCAT+ double positive population used for sorting. Because not all BONCAT positive viruses displayed subsequent SYBR staining, all events from double positives (BONCAT+, SYBR+) were separated into two populations: all double positives (P3 population) and double positives with high fluorescence in SYBR (P1 population). Both populations were collected separately.

A total of 320 single viral particles were sorted into 384 well plate (160 from population P1 and 160 from population P3), along with 8 pooled wells containing 2 wells with 10 events and 2 wells with 50 events each. Sorting was carried out at a flow rate below 1000 ev/sec, leaving the remaining wells empty as negative controls for multiple displacement amplification (MDA). Plates containing sorted samples were stored at -80 ºC until whole-genome amplification, which was performed following the same procedure described previously during 5h 30 min.

From the 384 well sorting plate, 13 wells showing successful amplification (12 single event wells and 1 pool containing  50  sorted  events) were selected for downstream PCR analysis (Supplementary Fig. 3D). PCR screening was performed to verify the presence of T7 phage DNA using primers targeting the T7 capsid gene 10.a [44], which amplify a 1,345 bp fragment of this gene. DNA extracted from T7 phage (9.7 ng/µL) was included as a positive control, H_2_O Mq as negative control, while a 1:20 dilution of the MDA amplified plate was used as the template for test samples. This dilution was chosen to approximate the initial DNA concentration of the positive control.

Each 25 µL PCR reaction contained 1× Taq polymerase buffer with standard MgCl₂ concentration, 0.2 mM of each dNTP, 0.5 µM of each primer, 1 U of Taq DNA polymerase (Thermo Scientific), and 1 µL of template DNA. The cycling protocol consisted of an initial denaturation at 94 °C for 3 min; 35 cycles of 94 °C for 30 s, 52.3 °C for 1 min , and extension to 72 °C for  2 min; and a final extension at 72 °C for 30 min. Amplification with the expected PCR product size was successfully observed in the pooled sample containing 50 sorted events confirming that we were able to recover indeed T7 phages.

**Fluorescence-Activated Viral BONCAT Sorting (FACS) using spectral cytometry**

Fluorescence-Activated Viral BONCAT Sorting (FACS) using spectral cytometry was performed on Mediterranean samples from an experimental BONCAT replicate collected on July 11, 2024, at Cape Huertas, Alicante. FACS analysis and sorting were conducted with a Cytek Aurora CS (Cytek) at the Center for Genomic Regulation (CRG/UPF) Flow Cytometry Unit (Barcelona, Spain), following experimental conditions consistent with those described above. Four liters of fresh surface seawater were collected; 2.5 L were amended with 10 µM HPG and incubated for 5 days with a 12 h:12 h light:dark photoperiod at 25 ºC, matching the average seawater temperature for that month. A control flask containing the same volume without HPG was processed in parallel. Post-incubation, 2 L of both samples (with and without HPG) were pre-filtered through 0.22 µm filters to remove cellular fractions and enrich with virus-like particles (VLPs) for FACS-based particle separation. The remaining 500 mL of each sample were processed without filtration to simultaneously detect active bacteria and BONCAT-positive VLPs. All samples underwent ultra-concentration and washing steps, culminating in a click chemistry reaction with 42 µL of click cocktail added to approximately 208 µL of sample according to the established BONCAT-FACS in-solution protocol described above, with oxygen removal by argon purging and a click reaction duration set to 6 hours for all samples. Post-click reaction, samples were washed three times with PBS 1X using 100 kDa Amicon filters and resuspended in 500 µL of 0.02 µm-filtered PBS. Prior to analysis in FACS, all instruments, reagents, and materials were subjected to strict DNA decontamination as described above for single-cell genomic technologies. Negative controls (PBS + BONCAT, seawater without HPG + BONCAT, and 0.22 µm filtered seawater + HPG) were employed to delineate BONCAT fluorescence thresholds enabling identification of bacterial and viral populations in both filtered and unfiltered samples. Spectral cytometry differs from conventional flow cytometry in measuring fluorescence across multiple detectors within each color channel (UV, blue, green-yellow, red), enabling precise identification of the AF-Picolyl-azide fluorescence peak detected at R2-H. Compared to negative controls, BONCAT-positive cell and VLP populations exhibited increased fluorescence peaks (Supplementary Fig. 8). Sorting by FACS targeted only BONCAT-positive viruses, sorted into two separate 96-well plates (filtered 0.22 µm and unfiltered samples after BONCAT incubation). Approximately 30,500 BONCAT-positive viruses from each sample were sorted into several pools at rates of 100 particles/well (x5), 1000 particles/well (x5), and 5000 particles/well (x5). Additionally, sheath fluid was sorted into a separate plate as a negative control for whole-genome amplification by multiple displacement amplification (MDA). Plates were stored at -80 ºC until MDA. Real-time MDA was performed similarly to the protocol described previously, lasting 4 hours for filtered and 7 hours 10 minutes for unfiltered samples. Amplified pools from each sample were combined into a single library and sent to Macrogen (South Korea) for Illumina Nextera XT library preparation followed by sequencing on a NovaSeq 6000 platform (150 x 2 PE; approximately 3 Gb per sample). Bioinformatic analysis of the cleaning reads was conducted with Kaiju [14] as described above, revealing enrichment of reads classified as viral.

**References**

1. Hatzenpichler R, Scheller S, Tavormina PL, Babin BM, Tirrell DA, Orphan VJ. *In situ*  visualization of newly synthesized proteins in environmental microbes using amino acid tagging and click chemistry. *Environ. Microbiol* **2014***;***16**, 2568–2590.

2. Hatzenpichler R, Connon SA, Goudeau D, Malmstrom RR, Woyke T, Orphan VJ. Visualizing in situ translational activity for identifying and sorting slow-growing archaeal−bacterial consortia. *Proc Natl Acad Sci USA* 2016;**113**.

3. Pasulka AL, Thamatrakoln K, Kopf SH, Guan Y, Poulos B, Moradian A *et al.* Interrogating marine virus‐host interactions and elemental transfer with BONCAT and nanoSIMS‐based methods. *Environmental Microbiology* 2018;**20**:671–92.

4. Samo TJ, Smriga S, Malfatti F, Sherwood BP, Azam F. Broad distribution and high proportion of protein synthesis active marine bacteria revealed by click chemistry at the single cell level. *Front Mar Sci* 2014;**1**, DOI: 10.3389/fmars.2014.00048.

5. Rinke C, Lee J, Nath N, Goudeau D, Thompson B, Poulton N *et al.* Obtaining genomes from uncultivated environmental microorganisms using FACS–based single-cell genomics. *Nat Protoc* 2014;**9**:1038–48.

6. Tijerina JC, Martinez-Hernandez F, Beilinson V, Finney O, Orphan VJ, Diamond RA. Preparation of Cuvette-Based Sorters for Sorting Submicron Microbial Cells and Viruses from Environmental and Biological Samples. *Current Protocols* 2025;**5**:e70176.

7. Stepanauskas R, Sieracki ME. Matching phylogeny and metabolism in the uncultured marine bacteria, one cell at a time. *Proc Natl Acad Sci USA* 2007;**104**:9052–7.

8. Stepanauskas R, Fergusson EA, Brown J, Poulton NJ, Tupper B, Labonté JM *et al.* Improved genome recovery and integrated cell-size analyses of individual uncultured microbial cells and viral particles. *Nat Commun* 2017;**8**:84.

9. Woyke T, Sczyrba A, Lee J, Rinke C, Tighe D, Clingenpeel S *et al.* Decontamination of MDA Reagents for Single Cell Whole Genome Amplification. *PLoS ONE* 2011;**6**:e26161.

10. Martinez-Hernandez F, Fornas O, Lluesma Gomez M, Bolduc B, De La Cruz Peña MJ, Martínez JM *et al.* Single-virus genomics reveals hidden cosmopolitan and abundant viruses. *Nat Commun* 2017;**8**:15892.

9. Garcia-Heredia I, Bhattacharjee AS, Fornas O, Gomez ML, Martínez JM, Martinez-Garcia M. Benchmarking of single-virus genomics: a new tool for uncovering the virosphere. *Environ Microbiol* 2021;**23**:1584–93.

12. Bolger AM, Lohse M, Usadel B. Trimmomatic: a flexible trimmer for Illumina sequence data. *Bioinformatics* 2014;**30**:2114–20.

13. Prjibelski A, Antipov D, Meleshko D, Lapidus A, Korobeynikov A. Using SPAdes De Novo Assembler. *Current Protocols in Bioinformatics* 2020;**70**:e102.

14. Menzel P, Ng KL, Krogh A. Fast and sensitive taxonomic classification for metagenomics with Kaiju. *Nat Commun* 2016;**7**:11257.

15. Ondov BD, Bergman NH, Phillippy AM. Interactive metagenomic visualization in a Web browser. *BMC Bioinformatics* 2011;**12**:385.

16. Guo J, Bolduc B, Zayed AA, Varsani A, Dominguez-Huerta G, Delmont TO *et al.* VirSorter2: a multi-classifier, expert-guided approach to detect diverse DNA and RNA viruses. *Microbiome* 2021;**9**:37.

17. Nayfach S, Camargo AP, Schulz F, Eloe-Fadrosh E, Roux S, Kyrpides NC. CheckV assesses the quality and completeness of metagenome-assembled viral genomes. *Nat Biotechnol* 2021;**39**:578–85.

16. Camargo AP, Roux S, Schulz F, Babinski M, Xu Y, Hu B *et al.* Identification of mobile genetic elements with geNomad. *Nat. Biotechnol.* 1–10 (2023).

19. Hyatt D, Chen G-L, LoCascio PF, Land ML, Larimer FW, Hauser LJ. Prodigal: prokaryotic gene recognition and translation initiation site identification. *BMC Bioinformatics* 2010;**11**:119.

20. Camargo AP, Nayfach S, Chen I-MA, Palaniappan K, Ratner A, Chu K *et al.* IMG/VR v4: an expanded database of uncultivated virus genomes within a framework of extensive functional, taxonomic, and ecological metadata. *Nucleic Acids Research* 2023;**51**:D733–43.

21. Buchholz HH, Michelsen ML, Bolaños LM, Browne E, Allen MJ, Temperton B. Efficient dilution-to-extinction isolation of novel virus–host model systems for fastidious heterotrophic bacteria. *The ISME Journal* 2021;**15**:1585–98.

22. Yang M, Xia Q, Du S, Zhang Z, Qin F, Zhao Y. Genomic Characterization and Distribution Pattern of a Novel Marine OM43 Phage. *Front Microbiol* 2021;**12**:651326.

23. Buchholz HH, Bolaños LM, Bell AG, Michelsen ML, Allen MJ, Temperton B. A Novel and Ubiquitous Marine Methylophage Provides Insights into Viral-Host Coevolution and Possible Host-Range Expansion in Streamlined Marine Heterotrophic Bacteria. *Appl Environ Microbiol* 2022;**88**:e00255-22.

24. Yang M, Du S, Zhang Z, Xia Q, Liu H, Qin F *et al.* Genomic diversity and biogeographic distributions of a novel lineage of bacteriophages that infect marine OM43 bacteria. *Microbiol Spectr* 2023;**11**:e04942-22.

25. Martinez-Hernandez F, Diop A, Garcia-Heredia I, Bobay LM, Martinez-Garcia M. Unexpected myriad of co-occurring viral strains and species in one of the most abundant and microdiverse viruses on Earth. *ISME J* 2022;**16**:1025–35.

26. Bolduc B, Jang HB, Doulcier G, You Z-Q, Roux S, Sullivan MB. vConTACT: an iVirus tool to classify double-stranded DNA viruses that infect *Archaea* and *Bacteria*. *PeerJ* 2017;**5**:e3243.

27. Arkin AP, Cottingham RW, Henry CS, Harris NL, Stevens RL, Maslov S *et al.* KBase: The United States Department of Energy Systems Biology Knowledgebase. *Nat Biotechnol* 2018;**36**:566–9.

28. Shannon P, Markiel A, Ozier O, Baliga NS, Wang JT, Ramage D *et al.* Cytoscape: A Software Environment for Integrated Models of Biomolecular Interaction Networks. *Genome Res* 2003;**13**:2498–504.

27. Roux S, Enault F, Ravet V, Pereira O, Sullivan MB*.* Genomic characteristics and environmental distributions of the uncultivated Far-T4 phages. *Front Microbiol* 2015;**6**.

30. Katoh K, Standley DM. MAFFT Multiple Sequence Alignment Software Version 7: Improvements in Performance and Usability. *Molecular Biology and Evolution* 2013;**30**:772–80.

31. Capella-Gutiérrez S, Silla-Martínez JM, Gabaldón T. trimAl: a tool for automated alignment trimming in large-scale phylogenetic analyses. *Bioinformatics* 2009;**25**:1972–3.

32. Kearse M, Moir R, Wilson A, Stones-Havas S, Cheung M, Sturrock S *et al.* Geneious Basic: An integrated and extendable desktop software platform for the organization and analysis of sequence data. *Bioinformatics* 2012;**28**:1647–9.

33. Roux S, Camargo AP, Coutinho FH, Dabdoub SM, Dutilh BE, Nayfach S *et al.* iPHoP: An integrated machine learning framework to maximize host prediction for metagenome-derived viruses of archaea and bacteria. *PLoS Biol* 2023;**21**:e3002083.

34. Rodriguez-R LM, Konstantinidis KT. The enveomics collection: a toolbox for specialized analyses of microbial genomes and metagenomes. 2016, DOI: 10.7287/peerj.preprints.1900v1.

35. Giovannoni SJ, Hayakawa DH, Tripp HJ, Stingl U, Givan SA, Cho J-C *et al.* The small genome of an abundant coastal ocean methylotroph. *Environ Microbiol* 2008;**10**:1771–82.

36. Huggett MJ, Hayakawa DH, Rappé MS. Genome sequence of strain HIMB624, a cultured representative from the OM43 clade of marine Betaproteobacteria. *Stand Genomic Sci* 2012;**6**:11–20.

37. Jimenez-Infante F, Ngugi DK, Vinu M, Alam I, Kamau AA, Blom J *et al.* Comprehensive Genomic Analyses of the OM43 Clade, Including a Novel Species from the Red Sea, Indicate Ecotype Differentiation among Marine Methylotrophs. *Appl Environ Microbiol* 2016;**82**:1215–26.

38. Layoun P, López-Pérez M, Haro-Moreno JM, Haber M, Thrash JC, Henson MW *et al.* Flexible genomic island conservation across freshwater and marine *Methylophilaceae*. *The ISME Journal* 2024;**18**:wrad036.

39. Rihtman B, Puxty RJ, Hapeshi A, Lee Y-J, Zhan Y, Michniewski S *et al.* A new family of globally distributed lytic roseophages with unusual deoxythymidine to deoxyuridine substitution. *Current Biology* 2021;**31**:3199-3206.e4.

40. Weinheimer AR, Brown JM, Thompson B, Leonaviciene G, Kiseliovas V, Jocys S *et al.* Single-particle genomics uncovers abundant non-canonical marine viruses from nanolitre volumes. *Nat Microbiol* 2025:1–13.

41. Thompson JD, Higgins DG, Gibson TJ. CLUSTAL W: improving the sensitivity of progressive multiple sequence alignment through sequence weighting, position-specific gap penalties and weight matrix choice. *Nucleic Acids Res* 1994;**22**:4673–80.

42. Besemer J, Borodovsky M. GeneMark: web software for gene finding in prokaryotes, eukaryotes and viruses. *Nucleic Acids Res* 2005;**33**:W451–4.

43. Brussaard CPD, Payet JP, Winter C, Weinbauer MG. Quantification of aquatic viruses by flow cytometry. In: Wilhelm S, Weinbauer M, Suttle C (eds.). *Manual of Aquatic Viral Ecology*. American Society of Limnology and Oceanography, 2010, 102–9.

44. Xu H, Bao X, Hong W, Wang A, Wang K, Dong H *et al.* Biological Characterization and Evolution of Bacteriophage T7-△holin During the Serial Passage Process. *Front Microbiol* 2021;**12**, DOI: 10.3389/fmicb.2021.705310.

45. Vila-Nistal, M., Martinez-Hernandez, F., Lluesma-Gomez, M., Fornas, O., Roux, S. and Martinez-Garcia, M., 2025. Limited consensus of marine viral diversity observed across techniques. *Environmental Microbiome*, *20*(1), p.157.

**Supplementary Figure 1. Stepwise workflow of the BONCAT-FACS protocol applied to seawater samples.** (1) BONCAT incubation: Control and HPG-amended samples (10 µM HPG) were incubated for 5 days under light:dark photoperiods. (2) Sample concentration and pre-click washing: Samples were concentrated using tangential flow filtration (TFF) and washed three times with 5 mL of 1X PBS (0.02 µm filtered) using 100 kDa Amicon filters (centrifuged at 2,500 xg for 3 min). After washing, the sample volume was adjusted to approximately 208 µL, containing HPG-incorporated active cells and viral progeny ready for BONCAT labelling. (3) BONCAT labelling and washing under anoxic click-reaction conditions: The click mix was prepared in two steps, then 208 µL of sample was mixed with 42 µL of click mix in 250 µL tubes, minimizing air space and flushed with argon. After the BONCAT reaction (6 h or 30 min), samples were washed using the same Amicon filters (+5 mL 1X PBS, centrifuged as before) to remove unbound dyes. HPG samples contain fluorescently labelled active cells and viral progeny, whereas controls (without HPG) include unlabelled viral progeny and active bacteria indistinguishable from inactive cells and pre-existing viruses. (4 and 5) Counterstaining and sorting: Pacific coast samples were counterstained with SYBR Gold and sorted by FACS using double thresholds (BONCAT+/SYBR+ for active cells and viral progeny; BONCAT−/SYBR+ for inactive cells and pre-existing viruses). Mediterranean coast samples were analyzed and sorted directly by FACS for BONCAT+ cells and viruses.

**Supplementary Figure 2. Flow cytometry gating strategy for sorting viral progeny and active cell populations (BONCAT+) in Pacific Ocean samples**. (A–C) Controls to define fluorescence thresholds and nonspecific background in green (SYBR Gold DNA stain for total cells and viruses) and red (AF647 Pycolyl-Azide for BONCAT+ active cells and viruses) channels: (A) PBS buffer without SYBR (sample washing and resuspension buffer). (B) PBS with SYBR-Gold, showing fluorescence in both SYBR and BONCAT channels. (C) Seawater sample without HPG after the click reaction, showing negligible BONCAT channel autofluorescence. These controls set thresholds to distinguish sample signal from background noise. (D) Flow cytometry plot of seawater sample with HPG, double stained with SYBR and BONCAT. SYBR fluorescence first identifies viral and cell populations (SYBR+). These are then visualized in the BONCAT channel to detect BONCAT+ subpopulations (cells SYBR+ and VLPs SYBR+). Identification of double-positive regions (SYBR+/BONCAT+) is based on comparison with the corresponding HPG-negative control (E). (E) HPG-negative seawater control plotted similarly for gate setting. Active cells gate (SYBR+/BONCAT+) includes events above BONCAT fluorescence threshold defined by negative control (control without HPG), comprising 83.5% of total SYBR+ cells. The inactive cells gate lies below this threshold. For viral progeny, gating is similarly defined by comparison of VLP SYBR+ populations in the BONCAT channel from HPG sample to control without HPG, distinguishing viral progeny (BONCAT+) from pre-existing viruses (BONCAT-). The viral progeny gate includes ~1.5% of the total viral population (SYBR+). (F) Flow cytometry plot of seawater sample with HPG analyzed in the BONCAT channel and quantification of percent of viral progeny. It is discriminate active cells comprise 2.13% of total events and VLPs BONCAT+ represent 9.94% of total events. To quantify the proportion of viral progeny (newly produced viruses), gate percentages were converted into absolute counts base on 100,000 total events. Not all BONCAT + viruses are SYBR+, so to estimate total VLPs abundance we account for overlap using the formula: (VLPs SYBR+) + (VLPs BONCAT+) − (VLPs SYBR+BONCAT+). This yields an estimated 16.5% of viral progeny among total VLPs.

**Supplementary Figure 3. Detection and identification of newly synthesized phages T7 from *E. coli* cultures in M9 by viral BONCAT-FACS**. (A) Confocal microscopy images (overlay of BONCAT and SYBR channels) showing new production of T7 phages after infection of *E. coli* cultures incubated with 100 µM HPG following overnight infection and a 30-minute click reaction using the standard viral BONCAT protocol with AF637-picolyl-azide (red fluorescence). The zoomed viral particle (VLP) reveals colocalization of BONCAT+ and SYBR+ signals. The control sample (*E. coli* culture in M9 without HPG incubation and infected with T7) shows SYBR+ labelling only and was used to set confocal microscope parameters. Both microscopy images were acquired on a Zeiss LSM 800 Confocal Scanning Microscope using 640 nm excitation laser for BONCAT signal (60% laser intensity, 650 V detector gain) and 488 nm excitation laser for SYBR (100% intensity, 650 V gain). To prevent channel bleed-through, staining was acquired in individual channels and digitally merged. Scale bar: 2 µm. (B) Flow cytometry plots of T7 samples from 30-minute standard click reaction and subsequent SYBR Gold counterstaining after fixation with 0.5% glutaraldehyde. PBS 1X buffer with click reaction, fixed and stained with 0.5% SYBR, served as BONCAT negative control to set fluorescence thresholds. The T7 phage sample without HPG (BONCAT-SYBR+) was first used to define BONCAT-negative fluorescence threshold on a BONCAT vs SSC-H plot, then thresholds for double positive events were established on a BONCAT vs SYBR plot. In the HPG-incubated T7 sample (BONCAT+SYBR+), the BONCAT+ population is visible on BONCAT vs SSC-H, while two double positive subpopulations were sorted (P3: all double positives; P1: double positives with high SYBR fluorescence). (C) Real-time multi displacement amplification curve for 13 sorted wells showing amplification over 5 h 30 min. Positive control: 10 ng lambda phage DNA; negative control: TE buffer U 16 h. The plotted well corresponds to a pool of 50 sorted events (P14). (D) 1% agarose gel electrophoresis (120 V, 45 min) of PCR products amplifying gene 10.a from 5 of 13 amplified wells (L22, P14, B2, F17, C23). 10 µL of PCR product plus 2 µL loading buffer were loaded. The gel image is cropped; the positive control sample (extracted T7 phage DNA) was run in lower lanes. Negative control: H_2_O Mq. Ladder: 5 µL GeneRuler 1 kb Plus DNA Ladder (Thermo Scientific). An expected band size (1,000-1,500 bp) is visible in P14 (50 sorted events), consistent with expected product size (1,345 bp). A nonspecific band is present in both positive control and sample lanes.

**Supplementary Figure 4. Taxonomic assignment of sequenced reads from active and inactive cell fractions.** Taxonomic classification was performed using the Kaiju program (see Methods). The figure highlights the most abundant bacterial groups, with their relative abundances expressed as a percentage of total bacterial reads shown in bold in the taxonomic graphs. The relative abundance of the OM43 clade is specifically indicated as a percentage of total Proteobacteria reads.

**Supplementary Figure 5. Genetic comparison of viruses recovered in our study with non-canonical DNA viruses of the Naomiviridae family that substitute deoxythymidine with deoxyuridine (dU).** (A) Reference sequence of the non-canonical Naomiviridae VC1099 shown in the alignment was obtained from [40]. Alignment of sequences similar to reference sequence VC1099 was performed with CLUSTAL W. Open reading frame (ORF) prediction was done using the Genmark tool, and annotation was carried out using BLAST conserved domain search and BLASTp. (B) Zoom of cluster 5 from the viral network, highlighting Roseobacter virus SIO1 as a representative reference genome. Viral BONCAT contigs similar to non-canonical Roseobacter phages are shown in yellow, with particular emphasis on contigs used in the alignment.

**Supplementary Figure 6. Fragment recruitment analysis of active (A) and non-active (B) cell reads from the Pacific Ocean against 13 OM43 genomes from isolated strains.** Sequenced reads from active and inactive cell fractions were mapped against a curated collection of 13 OM43 bacterial genomes. The strain *Methylophilales bacterium* H5P1 (highlighted in bold) was recovered in both active and non-active fractions, indicating its presence in diverse physiological states within the sampled microbial community.

**Supplementary Figure 7.** Eukaryote viral contigs recovered in the Pacific and the Mediterranean coastal samples identified as large double stranded DNA viruses within the 40 Nucleocytoviricota and putative Nucleocitoviricota-infecting Virophages from the different BONCAT fractions. A combination of geNomad analysis and BLASTp against IMG/VR 4.1 database was used to corroborate these taxonomic assignments. Contigs without a definitive taxonomic assignment are listed as unassigned.

**Supplementary Figure 8. Flow cytometry plots and sequencing data demonstrating the implementation of sorting of viral progeny (BONCAT+) populations in Mediterranean seawater samples using spectral flow cytometry.** An experimental replicate performed with spectral flow cytometry after 6 hours of click reaction confirms the reproducibility of the viral BONCAT-FACS method across different cytometers. (A) Flow cytometry plots of samples incubated with HPG and stained via BONCAT reveal distinct populations of BONCAT+ active cells and BONCAT+ VLPs in the HPG-incubated seawater compared to controls without HPG. In addition to natural seawater samples incubated with and without HPG, subsamples were pre-filtered through 0.22 µm filters to remove cells and enrich viral-like particles (VLPs) before performing BONCAT protocol. This pre-filtration stem aimed to enrich the sample in VLPs and simultaneously reduce the probability of coincident events during sorting. Both natural (unfiltered) and filtered (0.22 µm) samples exhibit detectable BONCAT+ VLP populations. (C) Taxonomic assignment of sequenced reads from BONCAT+ VLPs sorted via spectral flow cytometry. Taxonomic classification was performed using the Kaiju program, with the most abundant viral groups and their relative abundances expressed as a percentage of total viral reads.

**Supplementary Table 1:** Viral progeny ratio of the clusters identified in the viral network shown in Figure 4. The table shows the number of viruses identified as viral progeny (VP) and inactive viruses (VI) within each cluster. The viral progeny ratio was calculated using the formula (1) to account for differences in sequencing depth due to the initial sorting strategy. Only datasets from Pacific Ocean samples were included, as both inactive and active fractions were sorted.

| **Cluster** | **Active viruses (VP)** | **Inactive viruses (VI)** | **n**  **(active + inactive)** | **viral progeny ratio** |
| --- | --- | --- | --- | --- |
| Cluster Far-T4-like phages | 180 | 19 | 199 | 82.57 |
| Cluster 37-F6-like phages | 15 | 2 | 17 | 78.95 |
| Cluster 1 | 68 | 66 | 134 | 34.00 |
| Cluster 2 | 17 | 27 | 44 | 23.94 |
| Cluster 3 | 43 | 1 | 44 | 95.56 |
| Cluster 4 | 62 | 16 | 78 | 65.96 |
| Cluster 5 | 93 | 41 | 134 | 53.14 |

**Supplementary Table 2.** Active (viral progeny) and non-active viral contigs associated with the large double stranded DNA viruses, Nucleocytoviricota according to geNomad assignment. These putative eukaryote-infecting viruses were also recovered from the active cell fraction. Data was later corroborated by viral protein comparison against the high-confidence IMG/VR v4.1 viral database. Contigs detected as Mimiviridae by geNomad but without classification assignment by BLASTP are shown in blue.

| **Sample** | **Viral** | **Population^1^** | **Length** | **Virus** | **Taxonomy (geNomad assignment)** | **IMG/VR** | **IMG/VR** | **IMG/VR** | **IMG/VR** |
| --- | --- | --- | --- | --- | --- | --- | --- | --- | --- |
|  | **contig** |  |  | **Score^2^** |  | **Hits^3^** | **Hits taxonomy^4^**  **(Nucleoviricota)** | **Hits Taxonomy^4^ (Megaviricetes)** | **Hits Taxonomy^4^ (Family)** |
| Pacific Ocean | Contig 164 | VP | 8,390 | 0,9831 | Megaviricetes;Algavirales;Phycodnaviridae | 362 | 362 | 358 | 356 |
|  | Contig 182 | VP | 8,121 | 0,9736 | Megaviricetes;Algavirales;Phycodnaviridae | 283 | 283 | 282 | 280 |
|  | Contig 232 | VP | 7,363 | 0,9764 | Megaviricetes;Algavirales;Phycodnaviridae | 97 | 97 | 97 | 96 |
|  | Contig 234 | VP | 7,346 | 0,9828 | Megaviricetes;Algavirales;Phycodnaviridae | 176 | 176 | 176 | 175 |
|  | Contig 275* | VP | 6,978 | 0,9738 | Megaviricetes;Algavirales;Phycodnaviridae | 3 |  |  |  |
|  | Contig 809 | VP | 6,921 | 0,9672 | Megaviricetes;Algavirales;Phycodnaviridae | 343 | 343 | 343 | 341 |
|  | Contig 839 | VP | 6,781 | 0,9761 | Megaviricetes;Algavirales;Phycodnaviridae | 588 | 588 | 565 | 565 |
|  | Contig 316 | VP | 6,419 | 0,9799 | Megaviricetes;Algavirales;Phycodnaviridae | 53 | 53 | 53 | 53 |
|  | Contig 321 | VP | 6,361 | 0,9822 | Megaviricetes;Algavirales;Phycodnaviridae | 154 | 154 | 154 | 152 |
|  | Contig 986 | VP | 5,936 | 0,9824 | Megaviricetes;Algavirales;Phycodnaviridae | 420 | 420 | 419 | 417 |
|  | Contig 993 | VP | 5,893 | 0,9671 | Megaviricetes;Algavirales;Phycodnaviridae | 126 | 123 | 123 | 121 |
|  | Contig 1057 | VP | 5,512 | 0,9793 | Megaviricetes;Algavirales;Phycodnaviridae | 97 | 97 | 97 | 93 |
|  | Contig 396 | VP | 5,386 | 0,9821 | Megaviricetes;Algavirales;Phycodnaviridae | 201 | 201 | 201 | 198 |
|  | Contig 148 | VI | 11,660 | 0,9796 | Megaviricetes;Algavirales;Phycodnaviridae | 325 | 325 | 325 | 316 |
|  | Contig 188 | VI | 10,456 | 0,9812 | Megaviricetes;Algavirales;Phycodnaviridae | 183 | 182 | 182 | 173 |
|  | Contig 194 | VI | 10,355 | 0,9818 | Megaviricetes;Algavirales;Phycodnaviridae | 90 | 90 | 90 | 90 |
|  | Contig 332 | VI | 7,413 | 0,9697 | Megaviricetes;Algavirales;Phycodnaviridae | 63 | 63 | 63 | 61 |
|  | Contig 340 | VI | 7,237 | 0,9817 | Megaviricetes;Algavirales;Phycodnaviridae | 370 | 370 | 370 | 359 |
|  | Contig 425 | VI | 5,967 | 0,9180 | Megaviricetes;Algavirales;Phycodnaviridae | 10 | 10 | 10 | 10 |
|  | Contig 453 | VI | 5,546 | 0,9781 | Megaviricetes;Algavirales;Phycodnaviridae | 21 | 21 | 21 | 15 |
|  | Contig 479 | VI | 5,276 | 0,9815 | Megaviricetes;Algavirales;Phycodnaviridae | 79 | 79 | 79 | 78 |
|  | Contig 491 | VI | 5,110 | 0,9607 | Megaviricetes;Algavirales;Phycodnaviridae | 83 | 83 | 83 | 82 |
|  | Contig 175 | VI | 10,882 | 0,9815 | Megaviricetes;Imitervirales;Mimiviridae | 4 | 4 | 4 |  |
|  | Contig 38* | CA | 12,336 | 0,9785 | Nucleocytoviricota;unassigned | 5 |  |  |  |
|  | Contig 292 | CA | 6,117 | 0,9757 | Megaviricetes | 4 | 3 | 3 | 3 |
|  | Contig 284 | CA | 6,237 | 0,9830 | Megaviricetes;Algavirales;Phycodnaviridae | 281 | 279 | 279 | 278 |
|  | Contig 264 | CA | 6,468 | 0,9824 | Megaviricetes;Algavirales;Phycodnaviridae | 150 | 150 | 150 | 149 |
|  | Contig 265 | CA | 6,467 | 0,9819 | Megaviricetes;Algavirales;Phycodnaviridae | 17 | 17 | 17 | 16 |
|  | Contig 113 | CA | 8,999 | 0,9818 | Megaviricetes;Algavirales;Phycodnaviridae | 557 | 557 | 557 | 550 |
|  | Contig 167 | CA | 7,868 | 0,9816 | Megaviricetes;Algavirales;Phycodnaviridae | 227 | 227 | 223 | 219 |
|  | Contig 169 | CA | 7,826 | 0,9806 | Megaviricetes;Algavirales;Phycodnaviridae | 583 | 583 | 580 | 574 |
|  | Contig 210 | CA | 7,116 | 0,9799 | Megaviricetes;Algavirales;Phycodnaviridae | 451 | 451 | 451 | 447 |
|  | Contig 336 | CA | 5,629 | 0,9797 | Megaviricetes;Algavirales;Phycodnaviridae | 358 | 358 | 357 | 357 |
|  | Contig 350 | CA | 5,501 | 0,9736 | Megaviricetes;Algavirales;Phycodnaviridae | 447 | 447 | 447 | 422 |
|  | Contig 32 | CA | 13,324 | 0,9712 | Megaviricetes;Algavirales;Phycodnaviridae | 367 | 367 | 367 | 359 |
|  | Contig 186 | CA | 7,495 | 0,9497 | Megaviricetes;Algavirales;Phycodnaviridae | 236 | 236 | 236 | 233 |
|  | Contig 78 | CA | 10,051 | 0,7260 | Megaviricetes;Imitervirales;Mimiviridae | 1 | 1 | 1 | 1 |
| Mediterranean Sea | Contig 12 | VP | 16,033 | 0,9735 | Megaviricetes;Algavirales;Phycodnaviridae | 2 | 2 | 2 |  |
|  | Contig 13 | VP | 15,711 | 0,9726 | Megaviricetes;Pimascovirales;Iridoviridae | 4 | 4 | 4 |  |
|  | Contig 7 | VP | 26,621 | 0,9724 | Megaviricetes | 3 | 3 | 3 |  |
|  | Contig 3 | VP | 67,645 | 0,9071 | Megaviricetes | 2 | 2 | 2 |  |

1 Population. Viral genomes obtained from the different sorted populations, CA: Active cells, VP: Viral progeny, VI : Non-active viruses.

2 Virus-score. Obtained from geNomad viral identification

3 IMG/VR hits: Number of hits (80% Similarity, 90% hit coverage) with high-confident viral genomes from IMG/VR v4.1

4 IMG/VR Hits taxonomy: Number of hits with the same taxonomic level assignment compared to geNomad

* contigs hits with Bamfordvirae kingdom according to IMG/VR v4.1

**Supplementary Table 3.** Detection of small double stranded DNA virophages Active (viral progeny, VP) and non-active viral contigs (VI) from Preplasmiviricota according to geNomad assignment. Data was later corroborated by viral protein comparison against the high-confidence IMG/VR v4.1 viral database.

| **Sample** | **Viral** | **Population^1^** | **Length** | **Virus** | **Taxonomy (geNomad)** | **IMG/VR** | **IMG/VR** | **IMG/VR** | **IMG/VR** |
| --- | --- | --- | --- | --- | --- | --- | --- | --- | --- |
|  | **contig** |  |  | **Score^2^** |  | **Hits^3^** | **Hits taxonomy^4^**  **(Preplasmoviricota)** | **Hits Taxonomy^4^ (Maveriviricetes)** | **Hits Taxonomy^4^ (Family)** |
| Pacific Ocean | Contig 29* | VP | 14,029 | 0,9755 | Maveriviricetes;Priklausovirales;Lavidaviridae | 5 |  |  |  |
|  | Contig 217 | VP | 7,606 | 0,9816 | Maveriviricetes;Priklausovirales;Lavidaviridae | 9 | 6 | 5 | 5 |
|  | Contig 1012 | VP | 5,760 | 0,9759 | Maveriviricetes;Priklausovirales;Lavidaviridae | 177 | 94 | 81 | 27 |
|  | Contig 239* | VI | 9,134 | 0,9722 | Maveriviricetes;Priklausovirales;Lavidaviridae | 2 |  |  |  |

1 Population. Viral genomes obtained from the different sorted populations, VP: Viral progeny, VI : Non-active viruses.

2 Virus-score. Obtained from geNomad viral identification

3 IMG/VR hits: Number of hits (80% Similarity, 90% hit coverage) with high-confident viral genomes from IMG/VR v4.1

4 IMG/VR Hits taxonomy: Number with the same level of taxonomic assignment compared to geNomad

* contigs hits with Bamfordvirae kingdom according to IMG/VR v4.1

**Supplementary Data 1-10 legends**

**Supplementary Data 1. Viral progeny ratio per cluster in the viral network**

Detailed calculation of the viral progeny ratio for the clusters identified in the viral network. This table includes all viral contigs assigned to each of the seven clusters identified in the network. For each cluster, contigs are labelled as *reference* if they correspond to reference genomes from the vContact2 database, *VP* if they belong to the active (viral progeny) fraction, and *VI* if they belong to the inactive viral fraction. The value of *n* reflects the number of viral contigs from our samples (active + inactive) detected within each cluster. Clusters highlighted in red exhibit a high viral progeny ratio, while those in blue show a low viral progeny ratio. Within cluster 5, viral BONCAT contigs similar to non-canonical Naomiviridae DNA viruses are marked in magenta, and the reference genomes identified as the closest group are shown in yellow.

**Supplementary Data 2. Similarity of viral-BONCAT contigs to vSAG 37-F6 proteins**

Results of a BLASTp search using ORFs predicted from the vSAG 37-F6 genome (Prodigal v2.6.3) against all viral-BONCAT contigs. Two contigs share >16 proteins with vSAG 37-F6, with ~74–75% average amino acid identity.

**Supplementary Data 3. Predicted microbial hosts of Far-T4 phages**

Host predictions based on iPHoP using IMG/VR v4 genomes sharing ≥80% of proteins with Far-T4 phages. Flavobacteriales-affiliated genomes are highlighted in green**.**

**Supplementary Data 4. Taxonomic profile of bacterial samples at the order level**

Taxonomic classification using Kaiju against the NCBI nr_euk database. The table is collapsed at the order level, showing relative abundances as percentage of total reads (percent_total) and percentage of bacterial reads only (percent_bacteria). Flavobacteriales are highlighted in green; the most active bacterial orders in blue.

**Supplementary Data 5. BLASTn output of 1,630 viral-BONCAT *bona fide* contigs against three concatenated reference genomes of non-canonical Naomiviridae DNA viruses substituting deoxyuridine for deoxythymidine, including isolates DSS3_VP1, DSS3_PM1 and the viral cluster VC1099SCGC Capsule1_10222112.**

**Supplementary Data 6. BLASTp output of proteins predicted from 13 viral-BONCAT contigs with nucleotide similarity (72–80%) to the uncultivated viral cluster VC1099SCGC Capsule1_10222112, identified by BLASTn.**

**Supplementary Data 7: Taxonomic profile at the phylum level**

Taxonomic classification using Kaiju against the NCBI nr_euk database. The table is collapsed at the phylum level. The most abundant phylum in active fractions is highlighted in blue; in inactive fractions, in red.

**Supplementary Data 8. Taxonomic profile at the family level**

Taxonomic classification using Kaiju against the NCBI nr_euk database. The table is collapsed at the family level. The most abundant bacterial family in the inactive fractions is highlighted in red.

**Supplementary Data 9. Taxonomic profile at the species level**

Taxonomic classification using Kaiju against the NCBI nr_euk database. The table is collapsed at the species level. For species in the phylum Proteobacteria (formerly Pseudomonodota), an additional column shows their percentage within the phylum (percent_Pseudomonadota). OM43 clade species are highlighted in blue.

**Supplementary Data 10.OM43 phage identification**

BLASTp comparison of viral-BONCAT contigs against known OM43 phages (e.g., Melnitz, Venkman, MEP301, MEP401–402) and 99 related metagenomic viral genomes. Four contigs were closely related to Melnitz, known to infect OM43 strain H5P1.
