# Supplementary figures and images for: Illuminating the newly produced viruses within the virosphere with bioorthogonal noncanonical amino acid tagging and single-virus genomic sequencing technologies
Source: ISME Commun. 2026 Mar 6;6(1):ycag048. doi: 10.1093/ismeco/ycag048 (PMC13037479; doi:10.1093/ismeco/ycag048)

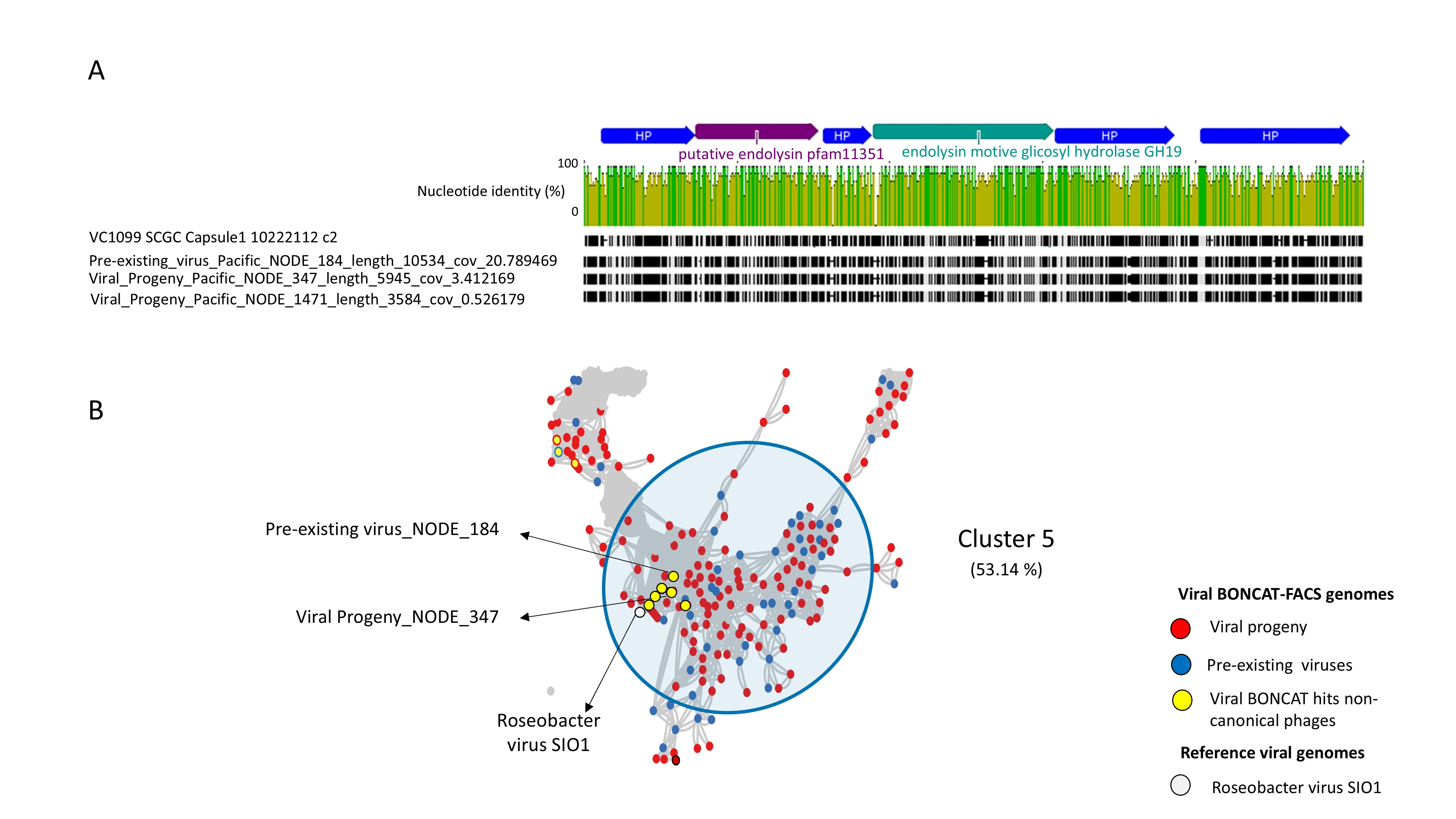

Supplement: ycag048_Fig_Supp_5 [file ycag048_fig_supp_5.jpeg]
